# NCX1 represents an ionic Na^+^ sensing mechanism in macrophages

**DOI:** 10.1371/journal.pbio.3000722

**Published:** 2020-06-22

**Authors:** Patrick Neubert, Arne Homann, David Wendelborn, Anna-Lorena Bär, Luka Krampert, Maximilian Trum, Agnes Schröder, Stefan Ebner, Andrea Weichselbaum, Valentin Schatz, Peter Linz, Roland Veelken, Jonas Schulte-Schrepping, Anna C. Aschenbrenner, Thomas Quast, Christian Kurts, Sabrina Geisberger, Karl Kunzelmann, Karin Hammer, Katrina J. Binger, Jens Titze, Dominik N. Müller, Waldemar Kolanus, Joachim L. Schultze, Stefan Wagner, Jonathan Jantsch

**Affiliations:** 1 Institute of Clinical Microbiology and Hygiene, University Hospital of Regensburg and University of Regensburg, Regensburg, Germany; 2 Department of Internal Medicine II, University Hospital of Regensburg and University of Regensburg, Regensburg, Germany; 3 Institute of Orthodontics, University Hospital of Regensburg, Regensburg, Germany; 4 Max Planck Institute of Biochemistry, Martinsried, Germany; 5 Institute of Radiology, University Hospital Erlangen, Friedrich-Alexander-Universität Erlangen-Nürnberg (FAU), Erlangen, Germany; 6 Department of Internal Medicine 4, University Hospital Erlangen, Erlangen, Germany; 7 Department for Genomics and Immunoregulation, Life and Medical Sciences (LIMES) Institute, University of Bonn, Bonn, Germany; 8 Department of Internal Medicine and Radboud Center for Infectious Diseases (RCI), Radboud University Medical Center, Nijmegen, the Netherlands; 9 Molecular Immunology and Cell Biology LIMES Institute, University of Bonn, Bonn, Germany; 10 Institute of Experimental Immunology, University of Bonn, Bonn, Germany; 11 Experimental and Clinical Research Center (ECRC), a cooperation of Charité-Universitätsmedizin Berlin and Max Delbruck Center for Molecular Medicine, Berlin, Germany; 12 Max Delbruck Center for Molecular Medicine in the Helmholtz Association (MDC), Berlin, Germany; 13 Institute of Physiology, University of Regensburg, Regensburg, Germany; 14 Department of Biochemistry and Genetics, La Trobe Institute for Molecular Science, La Trobe University, Bundoora, Australia; 15 Cardiovascular and Metabolic Disorders, Duke-NUS Medical School, Singapore; 16 Platform for Single Cell Genomics & Epigenomics at the German Center for Neurodegenerative Diseases (DZNE) and the University of Bonn, Bonn, Germany; Children's Hospital of Philadelphia and The University of Pennsylvania School of Medicine, UNITED STATES

## Abstract

Inflammation and infection can trigger local tissue Na^+^ accumulation. This Na^+^-rich environment boosts proinflammatory activation of monocyte/macrophage-like cells (MΦs) and their antimicrobial activity. Enhanced Na^+^-driven MΦ function requires the osmoprotective transcription factor nuclear factor of activated T cells 5 (NFAT5), which augments nitric oxide (NO) production and contributes to increased autophagy. However, the mechanism of Na^+^ sensing in MΦs remained unclear. High extracellular Na^+^ levels (high salt [HS]) trigger a substantial Na^+^ influx and Ca^2+^ loss. Here, we show that the Na^+^/Ca^2+^ exchanger 1 (NCX1, also known as solute carrier family 8 member A1 [SLC8A1]) plays a critical role in HS-triggered Na^+^ influx, concomitant Ca^2+^ efflux, and subsequent augmented NFAT5 accumulation. Moreover, interfering with NCX1 activity impairs HS-boosted inflammatory signaling, infection-triggered autolysosome formation, and subsequent antibacterial activity. Taken together, this demonstrates that NCX1 is able to sense Na^+^ and is required for amplifying inflammatory and antimicrobial MΦ responses upon HS exposure. Manipulating NCX1 offers a new strategy to regulate MΦ function.

## Abstract 2

Inflammation and infection can trigger local tissue accumulation of sodium ions, boosting pro-inflammatory antimicrobial activity of monocyte/macrophage-like cells. This study shows that the sensing of sodium ions by macrophages requires the sodium/calcium exchanger NCX1.

## Introduction

Infection and inflammation can trigger localized accumulation of sodium (Na^+^) in skin [1–3]. This accumulation is similar to that induced by high-Na^+^–containing diets and increases the effective osmolyte concentration within the skin to more than 40 mM above that of normal, isotonic blood [1,4].

Although blood Na^+^ is tightly regulated by the kidney, it has been established that monocyte/macrophage-like cells (MΦs) are necessary for the clearance of skin electrolytes upon dietary high-salt (HS) challenges [4,5]. However, the precise molecular mechanisms that orchestrate Na^+^ accumulation in the skin remain elusive, clearance by MΦs requires the osmoprotective transcription factor nuclear factor of activated T cells 5 (NFAT5) [4,6], which is the calcineurin-independent member of the NFAT family (reviewed in [7,8]).

In addition to regulating skin Na^+^ levels, the response of MΦs to infection and/or inflammation is modulated by increased local Na^+^ levels. Increases in Na^+^ limit anti-inflammatory regulatory MΦ activation while amplifying proinflammatory and antimicrobial activity [1,9–15]. We have shown that high Na^+^ increases NFAT5-dependent nitric oxide synthase 2 (*Nos2*) expression in lipopolysaccharide (LPS)-activated MΦs, resulting in increased antimicrobial nitric oxide (NO) production. High-Na^+^ conditions also enhanced autolysosome formation of infected MΦs, which together with increased NO, ultimately facilitates increased antimicrobial responses [1,10]. Although these findings established that alterations in local Na^+^ influence MΦ biology, the initial molecular responses of MΦs to high-Na^+^ environments remain unclear.

Here, we demonstrate that exposure to high extracellular Na^+^ stimulates the MΦ Na^+^/Ca^2+^ exchanger 1 (NCX1, also known as solute carrier family 8 member A1 [SLC8A1]), resulting in Na^+^ influx and Ca^2+^ efflux. We show that these NCX1-dependent currents are required for Na^+^-increased proinflammatory MΦ activity. Altogether, this study identifies a new molecule important for MΦs to sense increases in local Na^+^ levels and regulate their biology.

## Results

### Elevated extracellular Na^+^ triggers Na^+^ influx into MΦs

**Fig 1:**
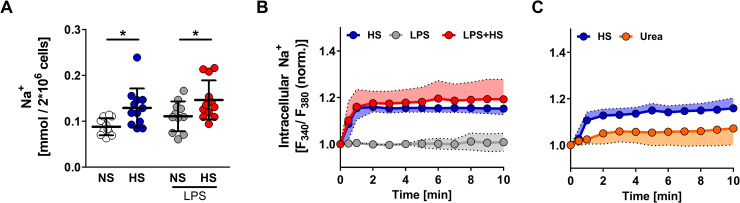
HS conditions induce rapid Na^+^ influx in MΦs. (A) Total Na^+^ content of RAW264.7 MΦs ± 10 ng/ml LPS under NS or HS (NS + 40 mM NaCl) conditions (mean ± SD; *n* = 11–12; Student *t* test ± Welch correction; *p < 0.05). (B) Relative [Na^+^]_i_ of RAW264.7 MΦs. Traces of RAW264.7 MΦs stimulated with HS, LPS, or both at t = 10 s (mean ± SD; *n* = 7–9). (C) Relative [Na^+^]_i_ of RAW264.7 MΦs. Traces of RAW264.7 MΦs stimulated with HS or 80 mM urea at t = 10 s (mean ± SD; *n* = 5). For numerical raw data, please see S1 Data. HS, high salt; LPS, lipopolysaccharide; MΦ, monocyte/macrophage-like cell; [Na^+^]_i_, intracellular Na^+^ in situ; NS, normal salt.

The elevated levels of extracellular skin Na^+^ can be mimicked in vitro by the addition of 40 mM NaCl to standard cell culture media [1,4,10,11]. We exposed MΦs to increased extracellular Na^+^ (+40 mM NaCl; +80 mOsm/kg; HS). Chemical analysis of Na^+^ content revealed that HS substantially increased cellular Na^+^ levels compared with normal salt (NS) in an LPS-independent manner (**Fig 1A**). To monitor intracellular Na^+^ in situ ([Na^+^]_i_), we used MΦs loaded with the Na^+^-sensitive dye sodium-binding benzofuran isophthalate (SBFI). LPS stimulation alone did not alter [Na^+^]_i_. HS exposure, however, resulted in an immediate increase in [Na^+^]_i_ in the absence or presence of LPS (**Fig 1B**). Of note, the osmolality control urea did not trigger substantial Na^+^ entry into MΦs (**Fig 1C**).

**Fig 2:**
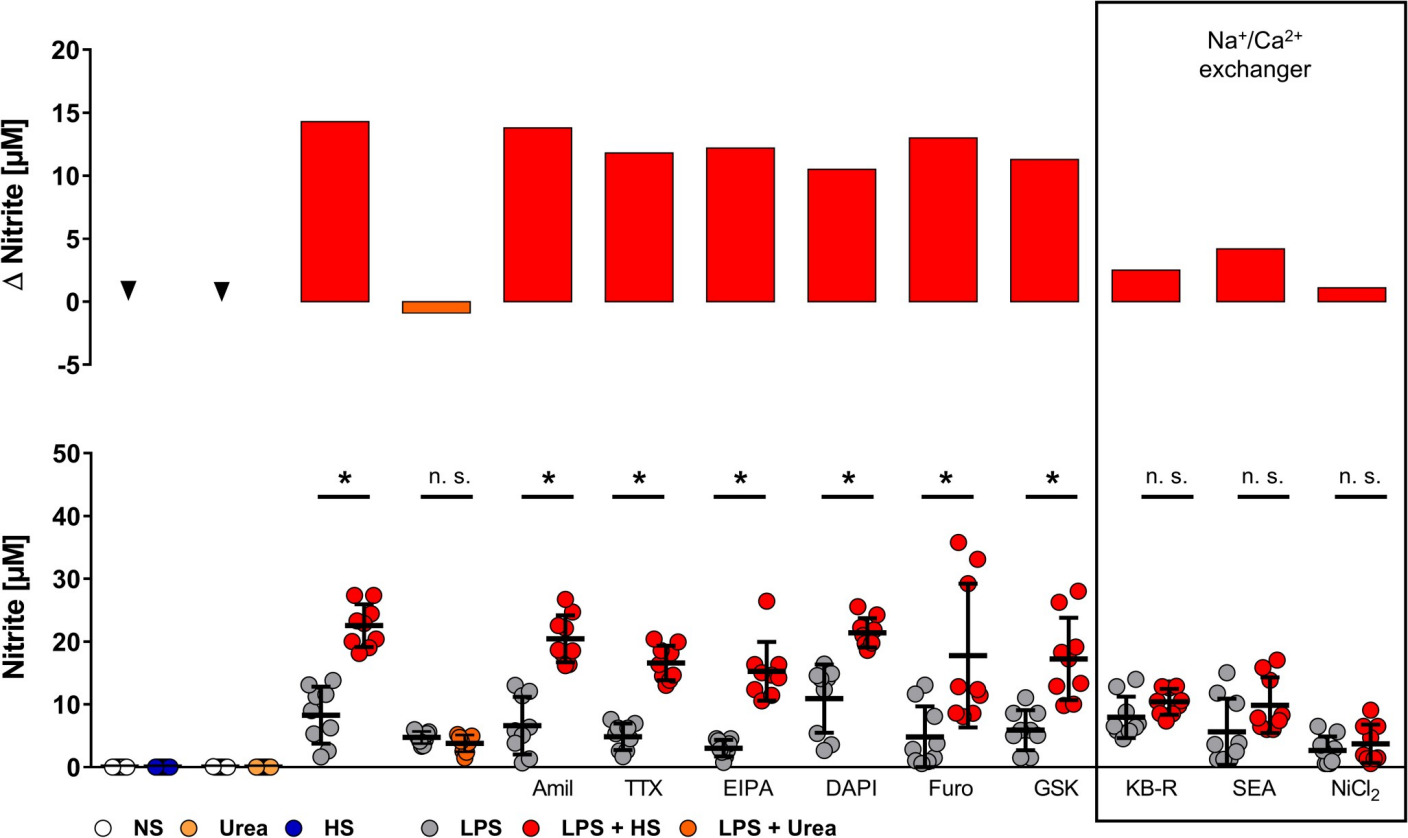
NCX inhibitors abrogate HS-boosted NO production of LPS-stimulated MΦs. Lower panel: nitrite levels of RAW264.7 MΦs pretreated with indicated inhibitors and stimulated ± LPS ± HS or 80 mM urea (mean ± SD; *n* = 9; Student *t* test or Mann–Whitney test; *p < 0.05). Upper panel: changes of means in nitrite production upon indicated stimulation and/or treatment (Δ nitrite). For numerical raw data, please see S1 Data. HS, high salt; LPS, lipopolysaccharide; MΦ, monocyte/macrophage-like cell; NCX, Na^+^/Ca^2+^ exchanger; NO, nitric oxide; NS, normal salt; n.s., not significant.

We hypothesized that one (or more) proteins at the plasma membrane might mediate HS-triggered Na^+^ influx. A query in the ImmGen database [16] revealed that mouse MΦs express transcripts for several ion channels, exchangers, and transporters, which are able to facilitate Na^+^ entry. This list includes the epithelial Na^+^ channel 1 alpha subunit, Na^+^/H^+^ antiporter, the voltage-gated Na^+^ channel, acid-sensing ion channels, Na^+^-K^+^-Cl^−^ symporter, and NCX1 (*Slc8a1*). In addition, HS-triggered signaling might also depend on transient receptor potential cation channel subfamily V member 4 (TRPV4). To measure whether Na^+^ influx by one of these molecules is involved in HS-boosted inflammatory (i.e., LPS-induced) MΦ activation, we used selective inhibitors targeting these molecules **(S1 Table**) and quantified NO production. Because HS alone (in the absence of LPS) does not induce NO production (**Fig 2**), as described earlier [1,10], we used MΦs stimulated with LPS in the presence of HS for this screening purpose (**Fig 2**). Conforming to our earlier findings [1,10], HS increased LPS-triggered NO release, whereas the osmolality control urea did not (**Fig 2**). The various inhibitors (**S1 Table**) revealed that only exposure to the NCX inhibitors KB-R7943 mesylate (KB-R) [17], SEA 0400 (SEA) [18], and NiCl_2_ [19] impaired HS-increased NO production in LPS-stimulated MΦs (**Fig 2**). Of note, these NCX inhibitors did not trigger NO production in the absence of LPS (**S1 Fig**).

**Fig 3:**
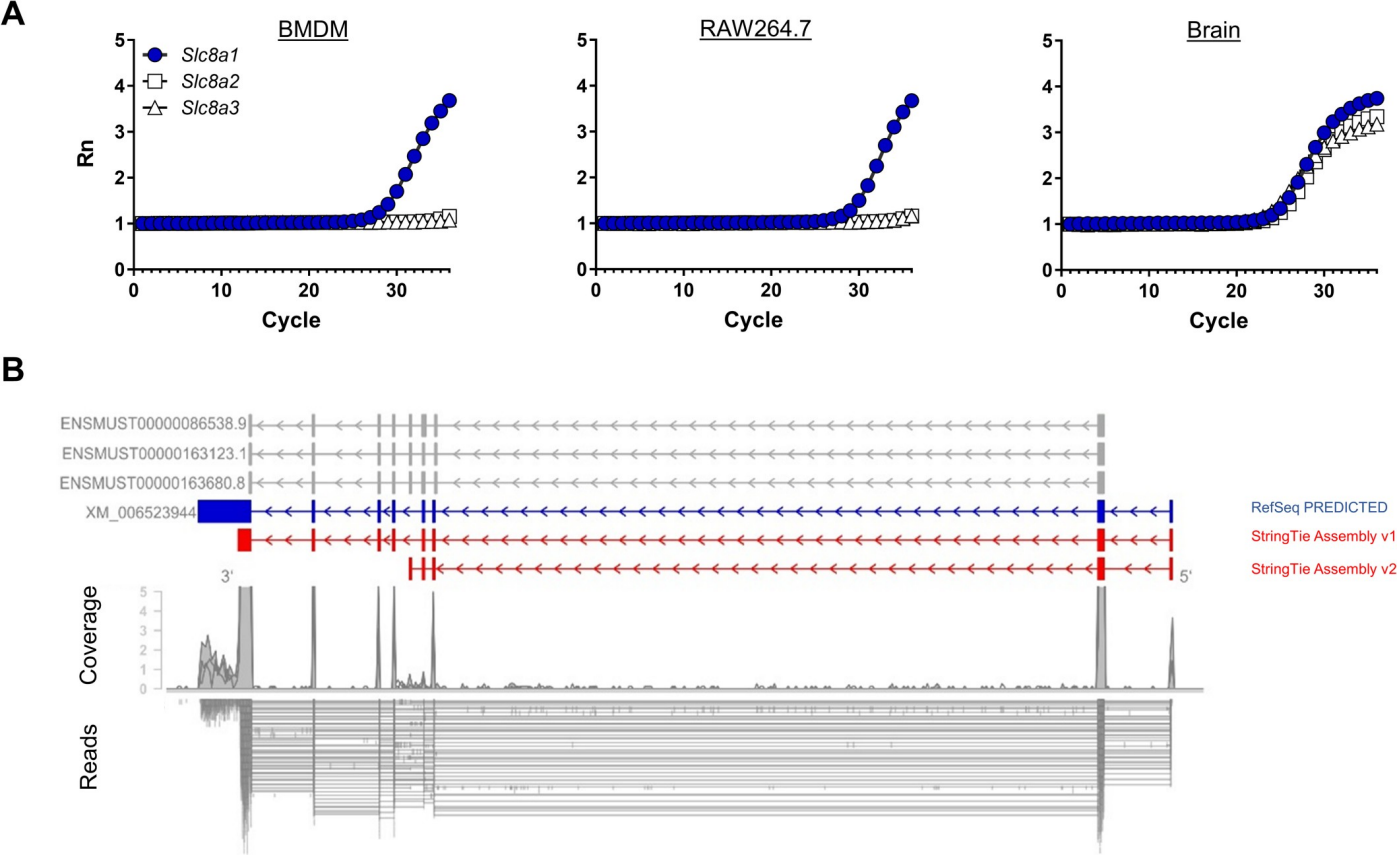
MΦs express a novel *Slc8a1* splice variant. (A) A representative real-time amplification plot of *Slc8a1*, *Slc8a2*, and *Slc8a3* in BMDMs, RAW264.7 MΦs, and brain tissue out of two experiments. (B) Shown are the alignments of RNA-seq reads of BMDMs and their coverage with annotated (gray; GENCODE: ENSMUST00000086538.9, ENSMUST00000163123.1, ENSMUST00000163680.8), predicted (blue; RefSeq PREDICTED: XM_006523944), and StringTie-assembled (red; StringTie Assembly version 1, StringTie Assembly version 2) *Slc8a1* splice variants. For numerical raw data, please see S1 Data. The RNA-seq data can be found in the GEO database (https://www.ncbi.nlm.nih.gov) under accession number GSE136662. BMDM, bone marrow–derived MΦ; GEO, Gene Expression Omnibus; MΦ, monocyte/macrophage-like cell; RNA-seq, RNA sequencing; *Slc8*, solute carrier family 8.

MΦs are known to express NCX [20], of which NCX1 and NCX3 are expressed in human MΦs [21]. A query of the ImmGen database [16] revealed that NCX1 (*Scl8a1*) is robustly expressed in mouse MΦs, whereas there was minimal expression of *Scl8a2* (the gene coding for NCX2) and *Scl8a3* (NCX3). These findings were confirmed by quantitative real-time PCR (qRT-PCR) analysis of mouse MΦs (**Fig 3A**). There are several *Slc8a1* splice variants known (reviewed in [22]). To determine the dominantly expressed variant (**Fig 3B**), we analyzed RNA sequencing (RNA-seq) data of bone marrow–derived MΦs (BMDMs). Transcriptome assembly identified two additional transcript variants (**Fig 3B**, red). Transcript abundance estimation revealed dominant expression of one novel variant (StringTie Assembly version 1), which skipped an internal exon and featured an additional 5′ exon upstream of the open reading frame (ORF), representing an alternative untranslated region (UTR). This novel *Slc8a1* variant has not been annotated in the standard GENCODE version M10 or RefSeq catalog, but it has been independently predicted by the National Center for Biotechnology Information (NCBI) eukaryotic gene prediction tool Gnomon under XM_006523944 (**Fig 3B**, blue).

### Increases in extracellular Na^+^ stimulate MΦ NCX currents

**Fig 4:**
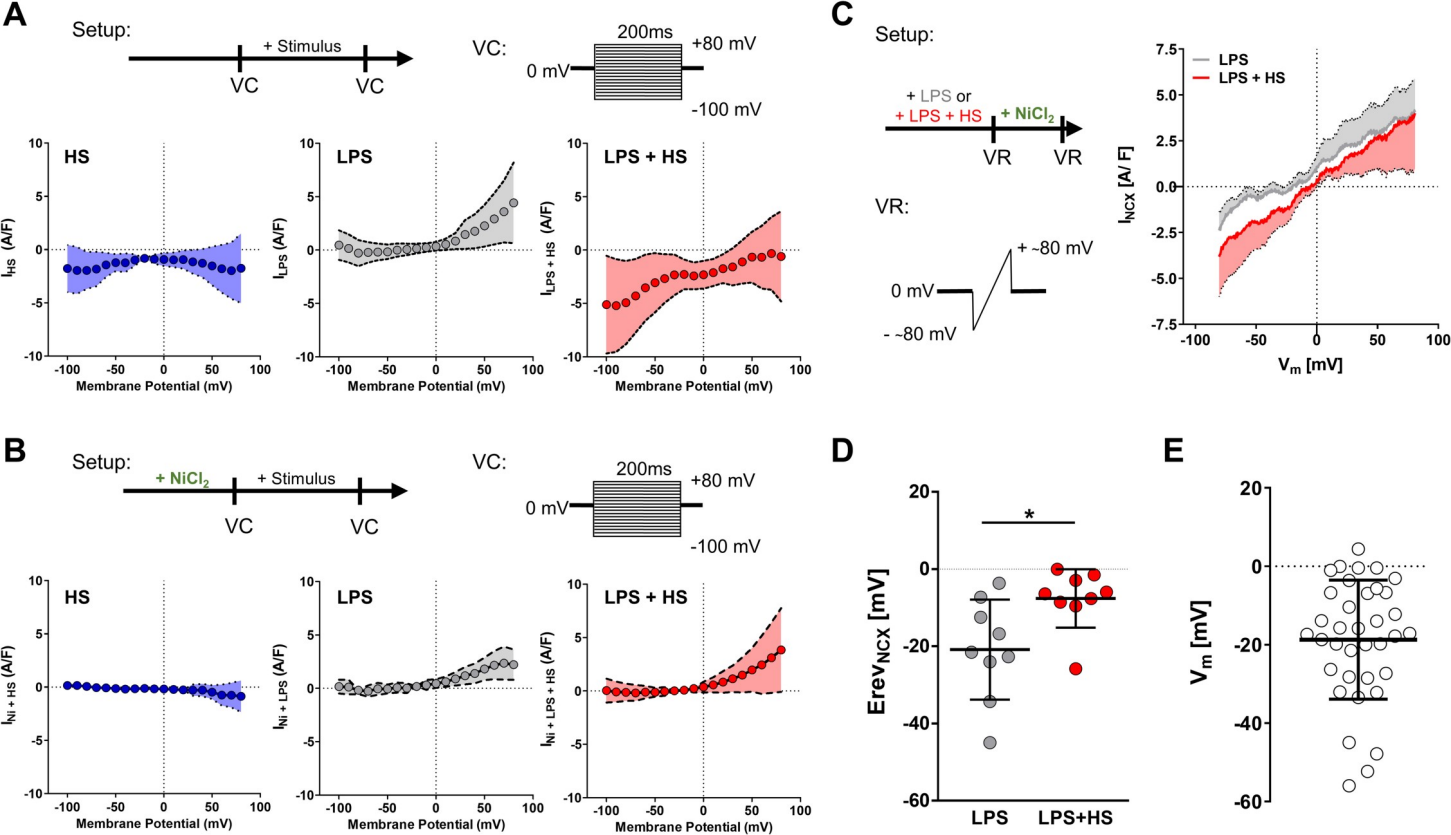
HS exposure results in NCX-mediated inward currents. (A) Current/voltage relationships of MΦ ± LPS ± HS. Whole-cell VC experiments were performed before and after stimulation of BMDMs. Voltage steps were applied, and differential currents (I_HS_, I_LPS_, I_LPS+HS_) were plotted (mean ± 95% CI; *n* = 10–13). (B) As in (A), but with NiCl_2_ pretreatment (means ± 95% CI; *n* = 10). (C) Current/voltage relationships of BMDMs stimulated with LPS ± HS followed by NiCl_2_ treatment. Whole-cell VR experiments were performed, and Ni-sensitive (i.e., NCX-sensitive) currents (I_NCX_) were determined (means ± 95% CI; *n* = 9). (D) Erev_NCX_ (means ± SD; *n* = 9; Mann–Whitney test; *p < 0.05). (E) Resting V_m_ of BMDMs (means ± SD; *n* = 36). For numerical raw data, please see S1 Data. BMDM, bone marrow–derived MΦ; Erev_NCX_, NCX reversal potential; HS, high salt; I, current; LPS, lipopolysaccharide; MΦ, monocyte/macrophage-like cell; NCX, Na^+^/Ca^2+^ exchanger; VC, voltage clamp; V_m_, membrane potential; VR, voltage ramp.

To explore the functional role of NCX in HS-increased inflammatory MΦ activation, we assessed whole-cell currents (whole-cell voltage clamp) of MΦ as a function of the membrane potential (V_m_). The HS-sensitive current, determined as the difference between the current in the presence and absence of HS, showed increased inward currents (**Fig 4A,** left panel), suggesting influx of positively charged ions upon HS exposure. As published earlier [23,24], we found that the LPS-sensitive current, determined as the difference between the current in the presence and absence of LPS, showed outward rectification at positive V_m_ (**Fig 4A,** middle panel). By contrast, exposure of MΦ to LPS in the presence of HS reduced outward currents and increased inward currents (**Fig 4A,** right panel). Overall, this is consistent with HS triggering the influx of positively charged ions at negative V_m_ and reduced net efflux at positive V_m_. In a separate set of experiments, we used NiCl_2_ to block NCX currents rapidly. NiCl_2_ pretreatment abolished HS-triggered inward currents (**Fig 4B**, left panel). However, NiCl_2_ treatment prior to LPS stimulation did not affect the outward rectification of the LPS-sensitive currents at positive V_m_ (**Fig 4B**, middle panel). Again, NiCl_2_ treatment prior to stimulation with LPS and HS abrogated HS-triggered increased inward currents but did not affect the LPS-sensitive currents at positive V_m_ (**Fig 4B,** right panel).

In order to analyze NCX currents directly, we measured the total current of LPS- and LPS + HS–treated MΦs before and after addition of NiCl_2_. The Ni-sensitive current, determined as the difference between the current in the absence and presence of NiCl_2_, was used as a direct measure of NCX current. Compared with LPS alone, at V_m_ values negative from the NCX reversal potential (Erev), additional HS exposure enhanced NCX inward current (**Fig 4C**). Altogether, this suggests that NCX mediates Na^+^ entry. Moreover, HS exposure shifted the Erev_NCX_ from −20.9 mV to −7.6 mV, consistent with the increased electrochemical gradient for Na^+^ ions upon HS (**Fig 4D**).

Since the forward mode of the NCX exchange activity can only occur at V_m_ negative of Erev [25], we measured the resting V_m_ in untreated and LPS-stimulated MΦs by whole-cell patch clamp technique (current clamp). In line with earlier publications [26–30], we detected a V_m_ of −18.7 mV in resting MΦs (**Fig 4E**), which did not significantly change upon LPS stimulation (**S2 Fig**). Under NS and LPS conditions, this resting V_m_ is very close to the Erev_NCX_. This incapacitates NCX-mediated currents to occur in LPS-treated MΦs under NS conditions; however, with HS exposure, it shifts the Erev into positive directions (**Fig 4D**), generating the driving force for forward-mode NCX inward currents to occur.

### Increases in extracellular Na^+^ result in Ca^2+^ loss

**Fig 5:**
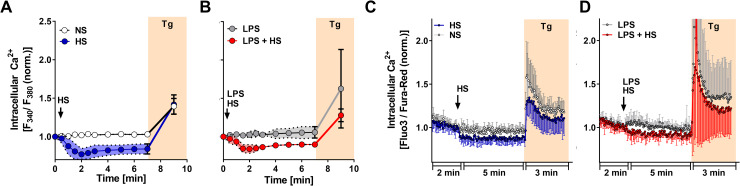
HS exposure causes Ca^2+^ loss. (A) Relative [Ca^2+^]_i_ levels of RAW264.7 MΦs. Traces of Fura-2–loaded RAW264.7 MΦs stimulated ± HS at t = 10 s (mean ± SD; *n* = 6). Where indicated, Tg was added (means ± SD; *n* = 2). (B) As in (A), but RAW264.7 MΦs were stimulated with LPS ± HS at t = 10 s (mean ± SD; *n* = 5). Where indicated, Tg was added (means ± SD; *n* = 2). (C) As in (A), but relative [Ca^2+^]_i_ levels were assessed in Fluo-3/Fura-Red–loaded MΦs (means ± SD; *n* = 6). (D) As in (B), but relative [Ca^2+^]_i_ levels were assessed in Fluo-3/Fura-Red–loaded MΦs (means ± SD; *n* = 8). For numerical raw data, please see S1 Data. [Ca2+]_i_, intracellular Ca^2+^ in situ; HS, high salt; LPS, lipopolysaccharide; MΦ, monocyte/macrophage-like cell; Tg, thapsigargin.

In its forward mode, NCX not only allows for Na^+^ entry but extrudes Ca^2+^ in exchange (reviewed in [22,31]). Accordingly, we hypothesized that a rapid efflux of Ca^2+^ from MΦs may accompany the HS-triggered increase in [Na^+^]_i_. Therefore, we analyzed the emitted fluorescence of Fura-2–loaded MΦs as a measure of intracellular Ca^2+^ in situ ([Ca^2+^]_i_) (**Fig 5A and 5B**). In accordance with previous publications [32–34], there was no long-lasting increase in [Ca^2+^]_i_ upon LPS stimulation under NS in MΦs (**Fig 5B**). Exposure to HS in the absence or presence of LPS stimulation, however, resulted in a rapid decrease of [Ca^2+^]_i_ (**Fig 5A and 5B**). We confirmed these results using flow cytometry–based analysis after staining with the Ca^2+^-sensitive dyes Fluo-3 and Fura-Red. Again, upon HS exposure in the absence of presence of LPS, we noted a rapid decrease of [Ca^2+^]_i_, which inversely mirrored the increase of [Na^+^]_i_ (**Fig 5C and 5D**). This further supports the notion that a single exchange mechanism may contribute to HS-triggered Na^+^ influx and Ca^2+^ loss in MΦs.

To assess whether HS-dependent decreases in [Ca^2+^]_i_ were mediated by increased uptake into intracellular stores, we exposed MΦs to thapsigargin (Tg, an inhibitor of the endoplasmic reticulum Ca^2+^-ATPase; reviewed in [35]) at the end of the experiments. Tg treatment resulted in a transient release of Ca^2+^ from intracellular stores, leading to an increase in [Ca^2+^]_i_ (**Fig 5A, 5B, 5C and 5D**). Importantly, HS exposure did not abolish the Tg-dependent Ca^2+^ release, suggesting that changes in Ca^2+^ uptake into intracellular stores upon HS exposure are not involved.

### Pharmacological inhibition of NCX interferes with HS-boosted MΦ function

**Fig 6:**
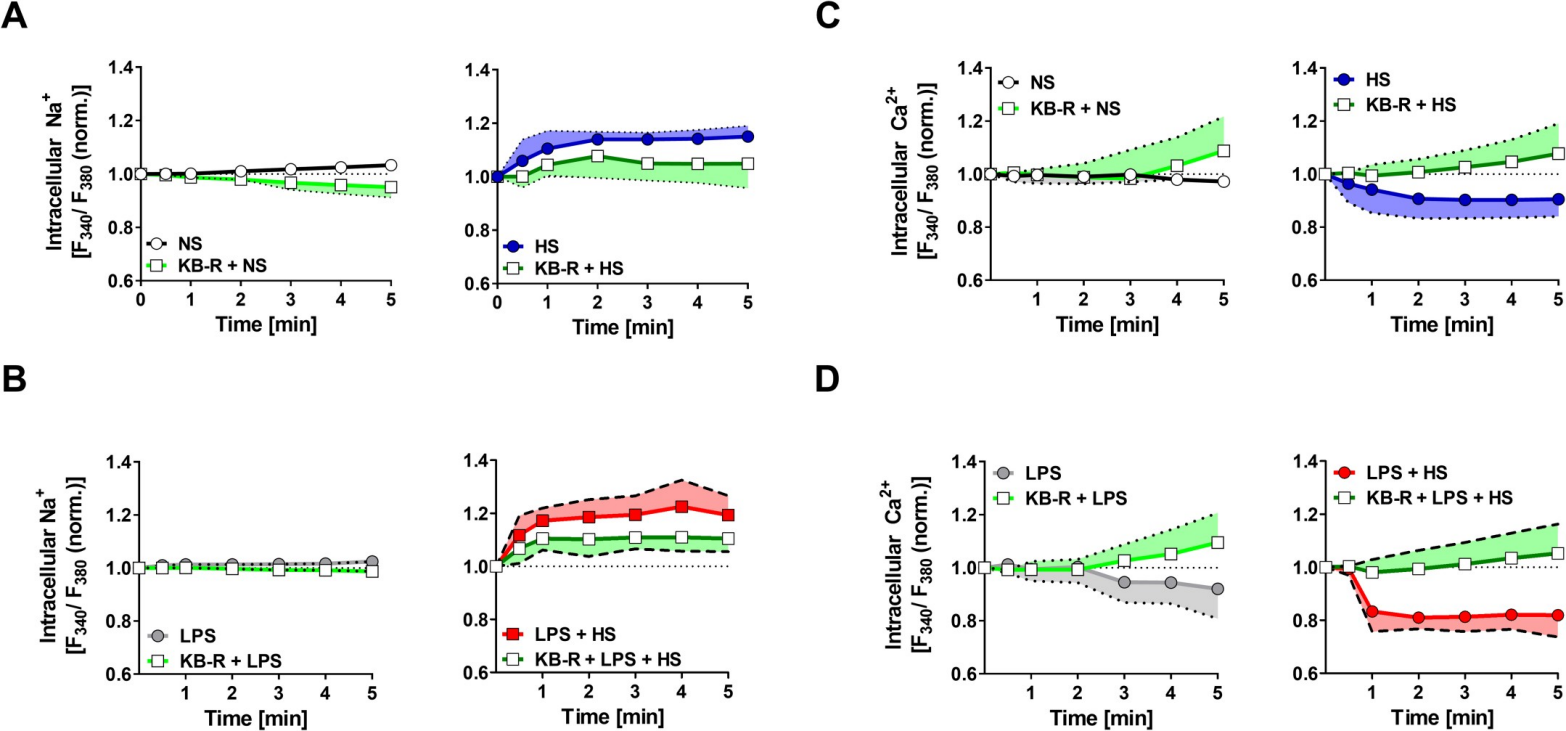
Pharmacological inhibition of NCX activity blunts HS-triggered Na^+^ influx and Ca^2+^ loss. (A) Relative [Na^+^]_i_ levels in RAW264.7 MΦs. Traces of RAW264.7 MΦs stimulated ± HS (at t = 10 s) ± KB-R pretreatment (means ± SD; *n* = 4–8). (B) Relative [Na^+^]_i_ levels in RAW264.7 MΦs. Traces of RAW264.7 MΦs stimulated with LPS ± HS (at t = 10 s) ± KB-R pretreatment (means ± SD; *n* = 5–8). (C) As in (A), but relative [Ca^2+^]_i_ levels were assessed in Fura-2–loaded MΦs (means ± SD; *n* = 5–8). (D) As in (B), but relative [Ca^2+^]_i_ levels were assessed in Fura-2–loaded MΦs (means ± SD; *n* = 6–8). For numerical raw data, please see S1 Data. [Ca^2+^]_i_, intracellular Ca^2+^ in situ; HS, high salt; LPS, lipopolysaccharide; MΦ, monocyte/macrophage-like cell; KB-R, KB-R7943 mesylate; [Na^+^]_i_, intracellular Na^+^ in situ; NCX, Na^+^/Ca^2+^ exchanger.

To further corroborate these findings, we used KB-R at a concentration known to inhibit the NCX forward mode (reviewed in [36]) and measured the HS-dependent changes in [Na^+^]_i_ and [Ca^2+^]_i_ in the absence or presence of LPS. KB-R treatment did not impair MΦ viability (**S3 Fig**) but blunted HS-triggered Na^+^ entry in the absence or presence of LPS (**Fig 6A and 6B).** This was paralleled by abrogated HS-mediated changes in [Ca^2+^]_i_ (**Fig 6C and 6D**).

**Fig 7:**
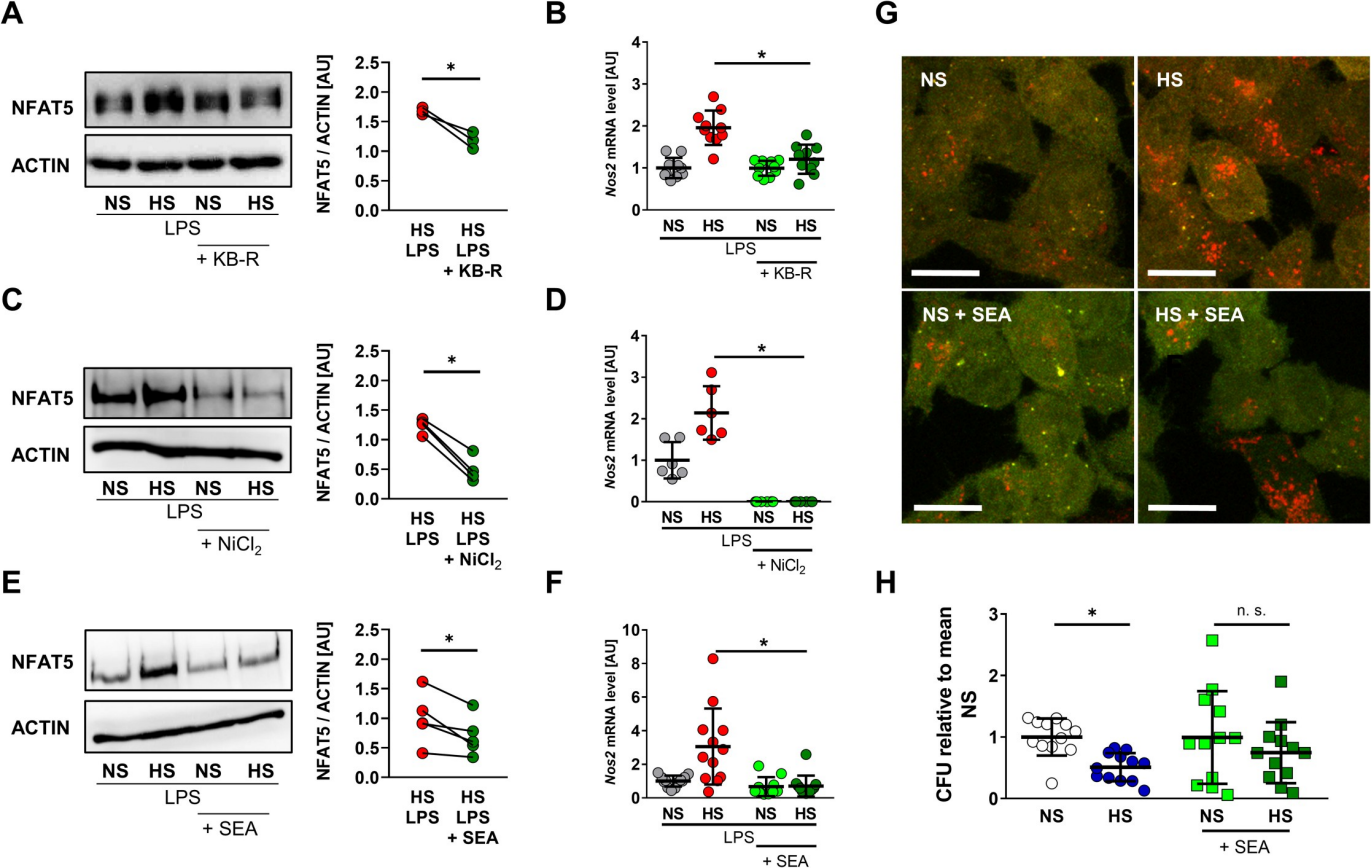
Pharmacological inhibition of NCX activity blocks HS-boosted MΦ activity. (A) Immunoblotting and densitometry of NFAT5 6 h after LPS ± HS in RAW264.7 MΦs ± KB-R pretreatment (*n* = 3; paired *t* test; *p < 0.05). (B) *Nos2* levels in RAW264.7 MΦs 4 h after LPS ± HS ± KB-R pretreatment (means ± SD; *n* = 10; Student *t* test; *p < 0.05). (C) Immunoblotting and densitometry of NFAT5 in RAW264.7 MΦs 4 h after LPS ± HS ± NiCl_2_ pretreatment (*n* = 4; paired *t* tests; *p < 0.05). (D) *Nos2* levels in RAW264.7 MΦs 4 h after LPS ± HS ± NiCl_2_ pretreatment (means ± SD; *n* = 6; Student *t* test + Welch correction; *p < 0.05). (E) Immunoblotting and densitometry of NFAT5 in RAW264.7 MΦs 4 h after LPS ± HS ± SEA pretreatment (*n* = 5; paired *t* test; *p < 0.05). (F) *Nos2* in RAW264.7 MΦs 4 h after LPS ± HS ± SEA pretreatment (means ± SD; *n* = 10–12; Mann–Whitney test; *p < 0.05). (G) RFP-GFP-mLC3 RAW264.7 MΦs were infected with *Escherichia coli* ± HS ± SEA pretreatment. Representative images 2 h after infection (RFP: red; GFP: green; scale bar: 10 μm). (H) Relative *E*. *coli* load at 2 h after infection of RAW264.7 MΦs ± HS ± SEA pretreatment (means ± SD; *n* = 12; Student *t* tests; *p < 0.05). For numerical raw data, please see S1 Data. For raw immunoblots, please see S1 Blots. CFU, colony forming unit; GFP, green fluorescent protein; HS, high salt; KB-R, KB-R7943 mesylate; LPS, lipopolysaccharide; mLC3, microtubule-associated protein 1 light chain 3; MΦ, monocyte/macrophage-like cell; NCX, Na^+^/Ca^2+^ exchanger; NFAT5, nuclear factor of activated T cells 5; *Nos2*, nitric oxide synthase 2; NS, normal salt; n.s., not significant; RFP, red fluorescent protein; SEA, SEA 0400.

Importantly, KB-R treatment interfered with HS-induced NFAT5 expression in the absence (**S4A Fig**) or presence of LPS stimulation (**Fig 7A**). In LPS-treated MΦs, this was paralleled by suppression of HS-boosted expression of the *Nfat5* target gene *Nos2* (**Fig 7B**), which is in line with the reduced effect of HS on LPS-induced NO production. (**Fig 2**). We additionally used NiCl_2_ and SEA to interfere with NCX activity. Again, these inhibitors did not affect MΦ viability (**S3 Fig**). NiCl_2_ treatment diminished HS-induced NFAT5 in the absence **(S4B Fig**) or presence of LPS (**Fig 7C)**. NiCl_2_ abolished HS-augmented expression of *Nos2* in LPS-stimulated MΦs (**Fig 7D**). Likewise, SEA interfered with HS-induced NFAT5 levels in the absence (**S4C Fig**) or presence of LPS (**Fig 7E**) and subsequently boosted *Nos2* expression (**Fig 7F**).

In addition to increasing *Nos2* expression, HS-induced *Nfat5* expression facilitates autolysosome formation upon infection, which is critically required for HS-boosted antibacterial activity directed against *E*. *coli* [10]. To test the impact of NCX inhibition on autolysosome formation, we used SEA in MΦs that express the tandem monomeric red fluorescent protein (RFP)-green fluorescent protein (GFP)–tagged microtubule-associated protein 1 light chain 3 (mLC3). Because GFP fluorescence is pH sensitive, GFP^+^RFP^+^ vesicles indicate autophagosomes, whereas GFP^−^RFP^+^ vacuoles mark degradative, acidified autolysosomes [37]. In accordance with our previous findings [10], HS exposure boosted the formation of autolysosomes in controls; however, in NCX-inhibited cells, HS failed to increase autolysosome formation, indicating disturbed autophagy (**Fig 7G**). Building on our earlier findings that HS-boosted antibacterial activity hinges on increased autophagy [10], NCX inhibition incapacitated the effect of HS on antibacterial activity (**Fig 7H**).

### Silencing of *Slc8a1* abrogates HS-amplified MΦ function

**Fig 8:**
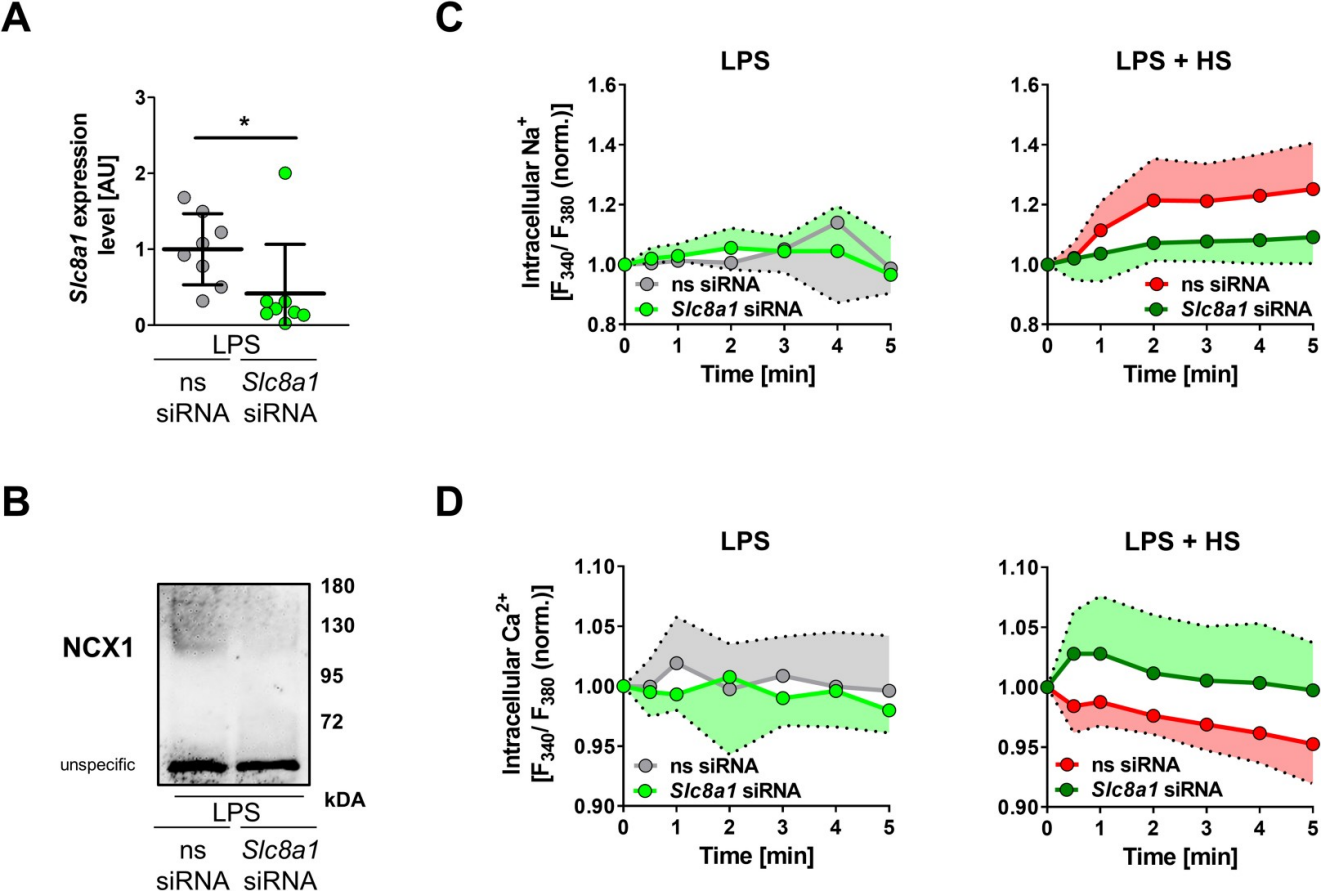
*Slc8a1* silencing decreases Na^+^ influx and Ca^2+^ efflux. (A) *Slc8a1* and (B) membranous NCX1 expression in LPS-stimulated ns or *Slc8a1*-specific siRNA–treated BMDMs after 4 h (means ± SD; *n* = 8; Mann–Whitney test; *p < 0.05). (C) Relative [Na^+^]_i_ levels of ns siRNA and *Slc8a1* siRNA–treated BMDMs exposed to LPS ± HS at t = 10 s (means ± SD; *n* = 5–7). (D) Relative [Ca^2+^]_i_ levels in ns siRNA and *Slc8a1* siRNA–treated BMDMs exposed to LPS ± HS at t = 10 s (means ± SD; *n* = 6–11). For numerical raw data, please see S1 Data. For raw immunoblots, please see S1 Blots. BMDM, bone marrow–derived MΦ; [Ca^2+^]_i_, intracellular Ca^2+^ in situ; HS, high salt; LPS, lipopolysaccharide; [Na^+^]_i_, intracellular Na^+^ in situ; ns, nonsilencing; NCX, Na^+^/Ca^2+^ exchanger; siRNA, small interfering RNA; S*lc8*, solute carrier family 8.

Next, we used RNA interference (RNAi) to target NCX1 expression in MΦs. Transfer of *Slc8a1-*specific small interfering RNA (siRNA) reduced *Slc8a1* mRNA levels in the absence (**S5 Fig**) and presence of LPS (**Fig 8A**). This was paralleled by diminished membranous expression of NCX1 (**Fig 8B**). Analysis of Ni-sensitive currents in *Slc8a1*-silenced MΦs upon HS exposure revealed that targeting *Slc8a1* diminished Ni-sensitive inward currents (**S6 Fig**). Because Ni-sensitive currents represent NCX currents, this suggests that silencing of *Slc8a1* is functional. Conforming to our pharmacological inhibitor data (**Fig 6**), silencing of *Slc8a1* strongly reduced HS-triggered increase in [Na^+^]_i_ (**Fig 8C**). Consistently, in this situation, HS failed to lower [Ca^2+^]_i_ (**Fig 8D**).

**Fig 9:**
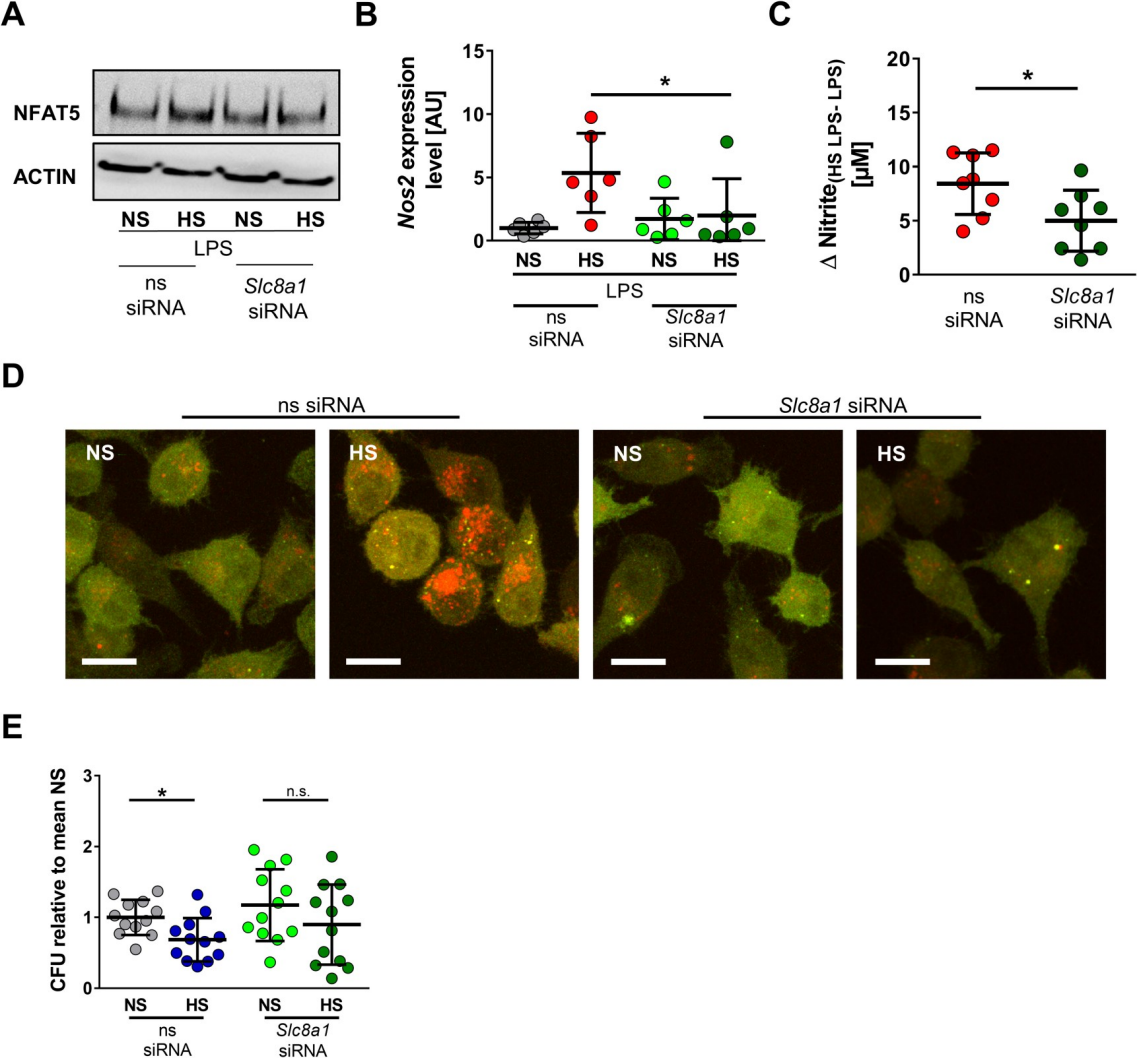
*Slc8a1* silencing abrogates HS-augmented MΦ activity. (A) ns siRNA and *Slc8a1* siRNA RAW264.7 were treated with LPS ± HS. Representative NFAT5 and ACTIN immunoblot 4 h after stimulation from two experiments. (B) As in (A), but *Nos2* in BMDMs 4 h after LPS ± HS (means ± SD; *n* = 6; Mann–Whitney test; *p < 0.05). (C) As in (B), but Δ nitrite_HS LPS-LPS_ after 24 h (means ± SD; *n* = 8; Student *t* test). (D) ns siRNA and *Slc8a1* siRNA–treated RFP-GFP-mLC3 RAW264.7 MΦs were infected with *E*. *coli* ± HS. Representative images 2 h after infection from three experiments (RFP: red; GFP: green; scale bar: 10 μm). (E) ns siRNA and *Slc8a1* siRNA–treated BMDMs were infected with *E*. *coli* ± HS. *E*. *coli* load at 2 h infection (means ± SD; *n* = 12; Student *t* tests; *p < 0.05). For numerical raw data, please see S1 Data. For raw immunoblots, please see S1 Blots. BMDM, bone marrow–derived MΦ; CFU, colony forming unit; GFP, green fluorescent protein; HS, high salt; LPS, lipopolysaccharide; mLC3, microtubule-associated protein 1 light chain 3; MΦ, monocyte/macrophage-like cell; NFAT5, nuclear factor of activated T cells 5; *Nos2*, nitric oxide synthase 2; NS, normal salt; ns, nonsilencing; n.s., not significant; RFP, red fluorescent protein; siRNA, small interfering RNA; *Slc8*, solute carrier family 8.

In line with this, *Slc8a1*-specific siRNA largely diminished the effect of Na^+^-induced NFAT5 protein levels in the absence (**S7A Fig**) and presence of LPS (**Fig 9A**). Silencing of *Slc8a1* under NS conditions did not induce NO production (**S7B Fig**). In LPS-activated MΦs, *Slc8a1* silencing abolished HS-triggered increases in *Nos2* mRNA expression (**Fig 9B**) and reduced HS-augmented NO production (**Fig 9C**). Finally, *Slc8a1* silencing abrogated HS-boosted autolysosome formation (**Fig 9D**) and subsequent antimicrobial activity (**Fig 9E**).

**Fig 10:**
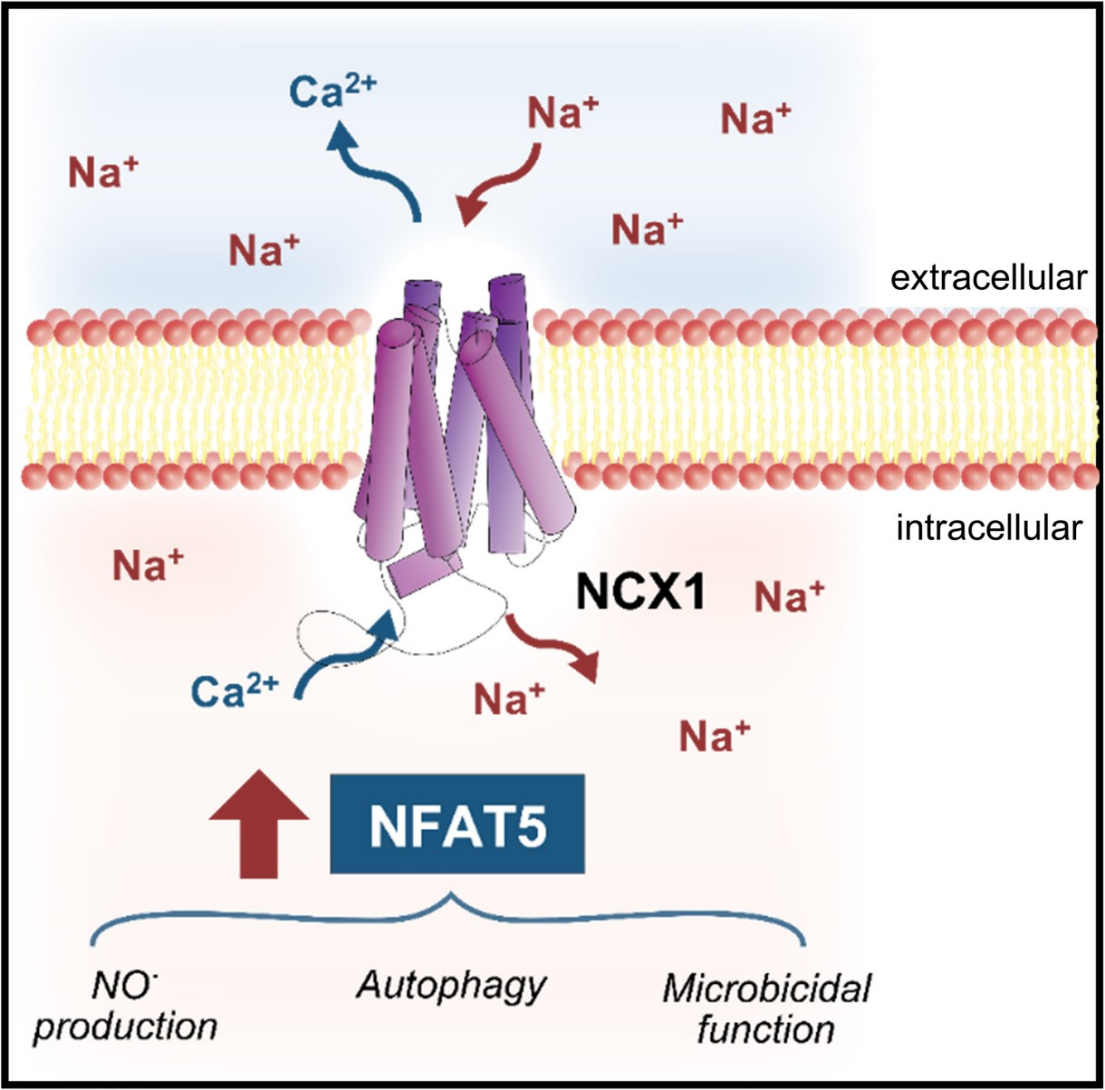
Graphical summary. NCX1 is required for Na^+^ entry and Ca^2+^ loss upon exposure of MΦs to HS conditions. In LPS-stimulated MΦs, NCX1 is required for HS-boosted NFAT5 accumulation and NO production. Moreover, NCX1 is required for enhanced autolysosome formation and bacterial removal upon HS exposure. HS, high salt; LPS, lipopolysaccharide; MΦ, monocyte/macrophage-like cell; NCX, Na^+^/Ca^2+^ exchanger; NFAT5, nuclear factor of activated T cells 5; NO, nitric oxide.

Overall, the RNAi experiments corroborate our pharmacological inhibition experiments and demonstrate that NCX1-dependent signaling is critical for HS-boosted inflammatory and antimicrobial response (**Fig 10**).

## Discussion

In addition to organic chemical signals, the local ionic inorganic tissue microenvironment is now recognized as a novel regulator of immune cell function (reviewed in [38–40]). We and others have demonstrated earlier that local Na^+^ imbalances are able to influence immune cell activation and to boost MΦ activation. HS amplification of MΦ activation hinges on signal molecules such as p38/mitogen-activated protein kinase, NFAT5, and mitochondrial reactive oxygen species (ROS) production, which play an important role in both adaption to osmotic stress and innate cell activation [12,41–45]. However, upstream LPS-independent mechanisms linking increased external Na^+^ to altered MΦ activation remained unknown.

In the present study, we show that exposure of MΦs to HS resulted in an immediate rapid increase in intracellular Na^+^. We investigated several Na^+^ entry pathways on their impact on HS-dependent MΦ stimulation. In line with earlier findings [15], we did not detect a contribution of voltage-gated Na^+^ channels. Moreover, our data demonstrate that amiloride-sensitive Na^+^ channels are not involved in HS-boosted MΦs. The latter have been implicated in microglia/MΦ function (reviewed in [46]) and Na^+^ sensing of dendritic cells [47]. In contrast, we show here that in MΦs, NCX1 contributes to Na^+^ entry and subsequent signaling that ultimately results in increased MΦ antimicrobial function upon HS exposure.

The increase of external Na^+^ from 140 mM to approximately 180 mM appears to result in a relatively small increase in the electrochemical gradient for Na^+^ across the membrane. Our data suggest that the plasma membrane of MΦs displays a high permeability for Na^+^ ions. Because the Erev_NCX_ of LPS-treated MΦs matched the V_m_ of resting and LPS-stimulated MΦs, NCX currents are incapacitated under NS conditions. By contrast, the small increase in the electrochemical Na^+^ gradient upon HS treatment shifts the Erev_NCX_ approximately 10 mV in the positive direction. Altogether, this facilitates the forward-mode activity of NCX, ultimately allowing Na^+^ cellular entry in exchange for Ca^2+^ efflux. Concordantly, we found a decrease in intracellular Ca^2+^. Considering the reported stoichiometric ratio of 3:1 for Na^+^/Ca^2+^ exchange (reviewed in [22]), a net movement of positive charges into the cell should rapidly depolarize the V_m_ of MΦs. Increases in intracellular Na^+^ and decreases in intracellular Ca^2+^ would rapidly reduce the driving force for NCX and incapacitate NCX currents again.

In accordance with others [32–34], we did not detect any long-lasting increases of [Ca^2+^]_i_ in MΦs exposed to LPS under NS, indicating that MΦs do not require such increases for their proinflammatory activity. This is in stark contrast to other cells, such as T cells (reviewed in [48]). Westphalen and colleagues reported that Ca^2+^ entry in alveolar MΦs through connexin 43–dependent interconnections from alveolar epithelial cells suppresses the inflammatory capacity of these MΦs [49]. Moreover, increases in intracellular Ca^2+^ can trigger adenosine monophosphate–activated protein kinase activity, which is able to curtail inflammatory properties of mononuclear myeloid cells [50]. This indicates that high Ca^2+^ levels can exert anti-inflammatory MΦ activity, implying that, conversely, lowering Ca^2+^ might lead to increased proinflammatory activity. Following this reasoning, we show that lowering [Ca^2+^]_i_ via NCX1 is linked to HS-boosted proinflammatory activation and antimicrobial MΦ function.

Moreover, low intracellular Ca^2+^ levels can induce autophagy (reviewed in [51]) where autophagy is regulated by numerous ion channels, exchangers, and transporters (reviewed in [52]). Previously, we found that the activation of autophagy represents an important auxiliary mechanism to boost antibacterial defense under HS conditions [10]. Here, we demonstrate that NCX1 plays an important role in this HS-boosted autophagy. Building on our earlier publication [10], our finding that interfering with NCX1 abolishes HS-increased autolysosomal formation provides a link between antibacterial responses and NCX1-dependent ion sensing. Our data show that extracellular Na^+^ is able to impact on intracellular organelle formation via NCX1. Very recently, it turned out that subcellular distribution of Na^+^ via two-pore channels plays an important role in controlling the volume of macropinosomes and, thereby, the cell size of MΦs [53]. Determining how increases in extracellular Na^+^ influence these subcellular processes requires further studies.

It is tempting to speculate that sensing of the ionic microenvironment represents a very ancient mechanism that allows animals to sense barrier dysfunction and consequently amplify antimicrobial and inflammatory MΦ responses. Here, we show that Na^+^ sensing by NCX1 represents an ionic mechanism important for MΦs to sense danger (**Fig 10**). The identification of this molecule opens new avenues to fine-tune MΦ immunobiology.

## Material and methods

### Ethics statement

Animal care and use followed the regulations of the German Animal Welfare Act. Mice were housed at Zentrale Tierlaboratorien (ZTL) der Universität Regensburg and kept under conditions approved by Umweltamt der Stadt Regensburg.

### Reagents and antibodies

The stimulations were performed with LPS from Sigma-Aldrich (*E*. *coli* O111:B4; #2630) and NaCl from Merck (#1.06400.1000). The channel inhibitors were purchased from Tocris (KB-R7943 mesylate [#12144] [17], SEA0400 [#6164] [18], amiloride [#0890] [54], EIPA [#3378] [55], and GSK 2193874 [#5106] [56]), Biotrend (tetrodotoxin [#BN0518] [57] and furosemide [(#BG0201] [58]), Sigma-Aldrich (DAPI [#D8417-5MG] [59]), and AppliChem (NiCl_2_ [#A2199,1000] [19]). For western blotting, we used rabbit anti-NCX1 antibodies from Abcam (#ab151608), rabbit anti-NFAT5 antibodies from Thermo Fisher Scientific (#PA1-023), and rabbit anti-ACTIN antibodies from Sigma-Aldrich (#A2066). Swine anti-rabbit HRP (Dako; #p0399) was used as secondary antibody.

### MΦ generation

We euthanized the mice before getting the marrow. BMDMs were generated in Teflon bags or Petri dishes supplemented with supernatant of L929 cells, as described earlier [60].

### RNAi in MΦ

Prior to the experiments, we confirmed that commercially available siRNA binds to the predicted *Slc8a1* transcript XM_006523944 using BLAST. We performed RNAi using *Slc8a1* siRNA (Dharmacon; # L-044925-00-0005) as described earlier [61]. After electroporation, cells were incubated for 1 d before further treatment and analysis.

### MΦ stimulation and infection experiments

MΦs were stimulated with LPS, resulting in a final concentration of 10 ng/ml LPS. NaCl (40 mM) or urea (80 mM) was added to MΦs where indicated. Both an increase of 40 mM in NaCl or adding of 80 mM urea increase osmolality by 80 mOsm/kg [1]. MΦ infection experiments were performed as described previously [1,10]. In brief, BMDMs or RAW264.7 MΦs were infected with *E*. *coli* HB101 (multiplicity of infection [MOI] of 100) for 1 h ± HS or SEA0400 (25 μM). MΦs were washed two times with PBS in order to remove remaining extracellular bacteria. Gentamicin (100 μg/ml) was added to the NS and HS conditions for an additional 1 h. Subsequently, cells were lysed with 0.1% Triton/0.05% Tween-20 in PBS. Bacterial solutions were serially diluted and plated on Müller–Hinton agar plates. The next day, colony forming units (CFUs) were manually counted and normalized to the mean of the untreated NS group.

### Measurement of intracellular Na^+^ by atomic absorption spectrometry

In all, 2 × 10^6^ RAW264.7 MΦs were stimulated with LPS ± HS for 10 min. Subsequently, cells were harvested and washed with iso-osmolal urea buffer. The MΦ pellet was lysed in 100 μl H_2_O_dd_ containing 0.1% Triton X, and the Na^+^ content was determined by atomic absorption spectrometry (Perkin Elmer, model 3100).

### Intracellular ion determination via epifluorescence microscopy

MΦs were seeded on FluoroDish plates. For Na^+^ and Ca^2+^ measurements, cells were stained as indicated with SBFI (Thermo Fisher Scientific; #S1264) or Fura-2 (Thermo Fisher Scientific; #F1221) in Tyrode solution (140 mM NaCl, 4 mM KCl, 1 mM MgCl_2_, 5 mM HEPES, 1 mM CaCl_2_, 10 mM glucose) containing 0.04% Pluronic (Sigma; #P2443). Cells were subjected to ratiometric quantification of the respective fluophor via live cell imaging for 5–10 min (epifluorescence microscope Motic model 410E). After 10 sec, 40 mM NaCl ± LPS was added to the dish, or cells were left untreated. Fluorescence emissions were recorded, analyzed using IonWizard (IonOptix Cooperation) as described earlier [62], and normalized to the start value.

### Intracellular Ca^2+^ measurements using flow cytometry

RAW264.7 MΦs were stained with the Ca^2+^-sensitive dyes Fluo-3 (Thermo Fisher Scientific; #F-1241) and Fura-Red (Thermo Fisher Scientific; #F-3021) [63] for 20 min at room temperature in Tyrode solution containing Pluronic. Subsequently, cells were washed and transferred into FACS tubes. Mean fluorescence intensities of Fluo-3 and Fura-Red were recorded over time using flow cytometry (FACSCanto II). After a lead-in phase of 2 min, HS ± LPS was added, or cells were left untreated. Samples were measured for an additional 5 min. Finally, Tg (Tocris; #1138) was added, and measurements were continued for 3 min. Recordings were analyzed via the “kinetics” tool of FlowJo (version 10). Fluo-3/Fura-Red ratios were calculated and normalized to the mean of the last three recordings before stimulation.

### Whole-cell patch clamp experiments

For electrophysiological measurements, BMDMs were used, and medium was replaced by an extracellular bathing solution (EC) containing 140 mM NaCl, 5 mM KCl, 2 mM CaCl_2_, 1 mM MgCl_2_, 10 mM HEPES, and 10 mM glucose (pH 7.35) and kept at room temperature. The dishes were placed on the stage of an inverted microscope. Patch pipettes were pulled from filamented borosilicate glass capillaries showing resistances of 2–4.5 MΩ when filled with intracellular pipette solution (IC) containing 140 mM K-gluconate, 5 mM NaCl, 2 mM MgCl_2_, 1 mM CaCl_2_, 11 mM EGTA, 10 mM HEPES, 2 mM Mg-ATP, and 0.3 mM Na-GTP (pH 7.35). The whole-cell configuration of the patch clamp technique [64] was used to record membrane currents and potential in voltage-clamp or current-clamp mode. Signals were recorded and digitized with an Axopatch 200B amplifier (Axon Instruments, Union City, CA) with a Digidata 1200 converter (Axon Instruments) or an EPC10 amplifier (HEKA Elektronik, Lambrecht/Pfalz, Germany). Analysis was performed using the software pCLAMP 10.2 (Axon Instruments) or PATCHMASTER 2x90 (HEKA).

Voltage steps were applied from a holding potential (−80 mV or −20 mV) from −100 mV to +80 mV in 10-mV increments and held for 200 ms. The voltage step protocol was performed 2 min after attaining the whole-cell configuration; afterward, cells were stimulated with 40 mM NaCl, 10 ng/ml LPS, or 5 mM NiCl_2_. At 3 min after stimulation, another voltage-clamp protocol was performed.

In a subset of experiments, a voltage ramp protocol from approximately −80 mV to approximately +80 mV (holding potential −20 mV, duration 4 sec, 40 mV/sec) was applied. Measured currents were normalized to the membrane capacitance. For V_m_ recordings, the cells were measured over 4 min in current-clamp-zero mode. After 50 sec, cells were stimulated. The first and last 50 sec of this recording were averaged to yield the V_m_ before and after stimulation.

For voltages between −40 mV and 30 mV, we fitted a straight line through the Ni-sensitive current data of LPS- and LPS + HS–treated MΦs using the nonlinear regression tool provided by GraphPad PRISM (version 6.0). We used the YIntercept and slope to determine Erev_NCX_.

### Autophagy characterization

We seeded RFP-GFP-mLC3 RAW264.7 MΦs (obtained from Invivogen; rawdf-mlc3) on coverslips and infected them with MOI 100 of *E*. *coli* HB101 ± HS ± 30 min preincubation with SEA0400. At 1 h after infection, cells were washed twice with PBS and incubated in NS or HS media containing 100 μg/ml gentamicin. At 2 h after infection, cells were washed, fixed in 4% paraformaldehyde for 20 min at room temperature, and mounted with ProLong Gold (containing DAPI). A Leica TCS SP5 confocal laser microscope was used for imaging. Images were processed using the Leica Application Suite (version 2.7.3.9723) and Microsoft PowerPoint.

### Bioinformatic analysis of *Slc8a1* isoform expression

RNA of three murine BMDM samples differentiated in vitro for 8 d using M-CSF were isolated with Trizol and the miRNeasy micro kit (Qiagen) according to the manufacturer’s protocol. The quality of the RNA was assessed by visualization of 28S and 18S band integrity on a Tapestation 2200 (Agilent). In all, 100 ng of RNA was converted into cDNA libraries using the TruSeq RNA library preparation kit version 2. Size distribution of cDNA libraries was measured using the Agilent High Sensitivity DNA assay on a Tapestation 2200 (Agilent). cDNA libraries were quantified using KAPA Library Quantification Kits (Kapa Biosystems). After cluster generation on a cBot, 75-bp single-read sequencing was performed on a HiSeq1500.

For alignment and genome-guided transcriptome assembly, we followed the “new tuxedo” protocol [65]. After base calling and demultiplexing using CASAVA version 1.8.2, the 75-bp single-end reads were aligned to the mouse reference genome mm10 using HISAT2 version 2.0.6 with option–dta and sorted and indexed using Samtools version 0.1.19. Subsequently, transcriptome assembly was performed using StringTie version 1.3.2d, with default parameters for each of the three samples guided by GENCODE vM10 transcriptome annotation. Merging the individual assemblies using the StringTie–merge option produced two novel multiexon transcript variants for *Slc8a1*, labeled StringTie Assembly version 1 and version 2 (**Fig 3B**). Transcript abundances were estimated by rerunning StringTie with options -B -e and analyzed using the R package Ballgown [66]. The RNA-seq data were uploaded in the Gene Expression Omnibus (GEO) database (https://www.ncbi.nlm.nih.gov) under accession number GSE136662.

### Quantification of nitrite and lactate dehydrogenase activity

Griess assays were used to quantify nitrite levels in the supernatants as described earlier [1,10]. The cell cytotoxicity kit (Roche; #11644793001) was used to measure lactate dehydrogenase (LDH) activities in supernatants and cell pellets. To assess the relative LDH release, the supernatant-to-pellet ratio of LDH activity was calculated.

### Immunoblotting

For analysis of NFAT5 and ACTIN, MΦs were lysed in 8 M urea containing a protease inhibitor cocktail (Roche; #11836170001), and immunoblotting was performed as described earlier [1,10]. For analysis of NCX1, membranes of MΦs were isolated using the Mem-PER Plus Kit (Thermo Fisher Scientific; #89842Y) according to the manufacturer’s instructions, and immunoblotting for NCX1 was performed. Images were acquired on an Intas Chemostar chemoluminescence imager and processed using Adobe Photoshop CS6 and Microsoft PowerPoint. We used ImageJ (version 1.50b; Rasband, W. S., ImageJ, United States National Institutes of Health, https://imagej.nih.gov/ij/) for densitometry of western blots.

### RNA isolation, reverse transcriptions, real-time PCR, and relative quantification

RNA isolation and qRT-PCR of cDNA was performed as described previously [10]. We obtained the following probes from Thermo Fisher Scientific for the analyses: *Slc8a1* (Mm01232254_m1), *Slc8a2* (Mm00455836_m1), *Slc8a3* (Mm01309304_m1), *Nos2* (Mm00440485_m1), and *Hprt* (Mm00446968_m1). Relative expression levels were determined using the ΔΔC_T_ method. *Hprt* served as endogenous control.

### Statistical analysis

All graphs were generated using GraphPad PRISM (version 6.0). Data sets were assessed for normality distribution via Kolomogorov–Smirnov tests. Normally distributed data were compared by unpaired, two-tailed Student *t* test (for two groups). We compared non-normally distributed data sets using Mann–Whitney tests (for two groups). In case of paired experiments (densitometry), we analyzed the data using paired Student *t* tests. Unless indicated otherwise, all data are depicted as means ± SD or 95% CI. We considered *p*-values < 0.05 as statistically significant.

## Supporting information

S1 Table Inhibitor data. List of used inhibitors and concentrations.
(DOCX)

S1 Fig Effects of NCX inhibitors on NO production. NO production was quantified 24 h after incubation of RAW264.7 MΦs ± HS ± after pretreatment with indicated inhibitor. (A) Inhibitor KB-R (*n =* 9). (B) Inhibitor NiCl_2_ (*n =* 6). (C) Inhibitor SEA (*n =* 6). For numerical raw data, please see S1 Data. HS, high salt; MΦ, monocyte/macrophage-like cell; KB-R, KB-R7943 mesylate; NCX, Na^+^/Ca^2+^ exchanger; NO, nitric oxide; SEA, SEA 0400.
(TIF)

S2 Fig Current clamp measurements of MΦ upon LPS stimulation. V_m_ of BMDMs before and after addition of 10 ng/ml LPS (*n =* 8; paired *t* test; *p < 0.05). For numerical raw data, please see S1 Data. BMDM, bone marrow–derived MΦ; LPS, lipopolysaccharide; MΦ, monocyte/macrophage-like cell; V_m_, membrane potential.
(TIF)

S3 Fig Effects on selected NCX inhibitors on MΦ viability. RAW264.7 MΦs were pretreated with indicated NCX inhibitors. At 4 h after stimulation ± LPS ± HS relative LDH release normalized to total cellular LDH content was assessed. Exposure of cells to 0.1% Triton X was used as positive control for cytotoxicity (means ± SD; *n =* 9). For numerical raw data, please see S1 Data. HS, high salt; LPS, lipopolysaccharide; MΦ, monocyte/macrophage-like cell; NCX, Na^+^/Ca^2+^ exchanger.
(TIF)

S4 Fig Effects of NCX inhibitors on NFAT5 accumulation. (A) NFAT5 levels 6 h after incubation of RAW264.7 MΦs ± HS ± KB-R pretreatment. Immunoblotting and densitometry (*n* = 4; paired *t* tests; *p < 0.05). (B) NFAT5 levels 4 h after incubation of RAW264.7 MΦs ± HS ± NiCl_2_ pretreatment. Immunoblotting and densitometry (*n* = 4; paired *t* tests; *p < 0.05). (C) As in (B), but SEA was used to inhibit NCX (*n* = 7; paired t tests; *p < 0.05). For numerical raw data, please see S1 Data. For raw immunoblots, please see S1 Blots. HS, high salt; KB-R, KB-R7943 mesylate; MΦ, monocyte/macrophage-like cell; NCX, Na^+^/Ca^2+^ exchanger; NFAT5, nuclear factor of activated T cells 5; SEA, SEA 0400.
(TIF)

S5 Fig *Slc8a1* silencing reduces *Slc8a1* mRNA levels. *Slc8a1* expression in ns or *Slc8a1*-specific siRNA–treated BMDMs (means ± SD; *n =* 8; Student *t* test; *p < 0.05). For numerical raw data, please see S1 Data. BMDM, bone marrow–derived MΦ; ns, nonsilencing; siRNA, small interfering RNA; *Slc8*, solute carrier family 8.
(TIF)

S6 Fig *Slc8a1* silencing diminishes inward currents upon HS treatment. Current/voltage relationships of BMDMs treated with ns siRNA and *Slc8a1* siRNA. These MΦ were stimulated with HS followed by NiCl_2_ treatment. Whole-cell VR experiments were performed, and Ni-sensitive (i.e., NCX-sensitive) currents were determined (means ± 95% CI; *n =* 9–10). For numerical raw data, please see S1 Data. BMDM, bone marrow–derived MΦ; HS, high salt; MΦ, monocyte/macrophage-like cell; NCX, Na^+^/Ca^2+^ exchanger; ns, nonsilencing; siRNA, small interfering RNA; *Slc8*, solute carrier family 8; VR, voltage ramp.
(TIF)

S7 Fig *Slc8a1* silencing reduces HS-augmented NFAT5 accumulation. (A) NFAT5 expression 4 h after LPS ± HS in ns siRNA and *Slc8a1* siRNA–treated RAW264.7 MΦs. Representative NFAT5 and ACTIN immunoblot out of two similar experiments. (B) As in (A), but HS-triggered Δ nitrite after 24 h (*n =* 3). For numerical raw data, please see S1 Data. For raw immunoblots, please see S1 Blots. HS, high salt; LPS, lipopolysaccharide; MΦ, monocyte/macrophage-like cell; NFAT5, nuclear factor of activated T cells 5; ns, nonsilencing; siRNA, small interfering RNA; *Slc8*, solute carrier family 8.
(TIF)

S1 Data Numerical raw data. All numerical raw data are combined in a single Excel file “S1_Data.xlsx”. This file consists of several spreadsheets. Each spreadsheet contains the raw data of one subfigure.
(XLSX)

S1 Blots Raw images. The file “S1_Blots.pdf” covers all uncropped western blot images, including size standards and descriptions.
(PDF)

## Abbreviations

BMDM: bone marrow–derived MΦ
[Ca^2+^]_i_: intracellular Ca^2+^ in situ
Erev: reversal potential
GFP: green fluorescent protein
HS: high salt
KB-R: KB-R7943 mesylate
LPS: lipopolysaccharide
MΦ: monocyte/macrophage-like cell
mLC3: microtubule-associated protein 1 light chain 3
[Na^+^]_i_: intracellular Na^+^ in situ
NCBI: National Center for Biotechnology Information
NCX: Na^+^/Ca^2+^ exchanger
NFAT5: nuclear factor of activated T cells 5
NO: nitric oxide
NS: normal salt
ns: nonsilencing
NOS2: nitric oxide synthase 2
ORF: open reading frame
qRT-PCR: quantitative real-time PCR
RFP: red fluorescent protein
RNAi: RNA interference
RNA-seq: RNA sequencing
ROS: reactive oxygen species
SBFI: sodium-binding benzofuran isophthalate
SEA: SEA 0400
siRNA: small interfering RNA
SLC8: solute carrier family 8
Tg: thapsigargin
TRPV4: transient receptor potential cation channel subfamily V member 4
UTR: untranslated region
V_m_: membrane potential.
