# Supplementary material for: NCX1 represents an ionic Na+ sensing mechanism in macrophages
Source: PLoS Biol. 2020 Jun 22;18(6):e3000722. doi: 10.1371/journal.pbio.3000722 (PMC7307728; doi:10.1371/journal.pbio.3000722)
Supplement: S1 Table — List of used inhibitors and concentrations. (DOCX) [file pbio.3000722.s001.docx]

| **Channel/ Transporter/**  **Exchanger** | **Abbreviation** | **Inhibitor** | **Inhibitor Abbreviation** | **Concentration**  **used** | **Reference** |
| --- | --- | --- | --- | --- | --- |
| Epithelial Na^+^ channel | ENaC | Amiloride | Amil | 100 µM | [54] |
| Na^+^ voltage-gated channel | Na_V_ | Tetrodotoxin | TTX | 10 µM | [57] |
| Na^+^/ H^+^ exchanger | NHE | Ethyl-isopropyl amiloride | EIPA | 5 µM | [55] |
| Acid-sensing ion channels | ASIC | 4',6-diamidino-2-phenylindole | DAPI | 10 µM | [59] |
| Na^+^-K^+^-Cl^-^ symporter | NKCC | Furosemide | Furo | 250 µM | [58] |
| Transient receptor potential vanilloid cation channel 4 | TRPV4 | GSK2193874 | GSK | 200 nM | [56] |
| Na^+^/ Ca^2+^ exchanger | NCX | KB-R7943 mesylate | KB-R | 20 µM | [17] |
| Na^+^/ Ca^2+^ exchanger | NCX | SEA0400 | SEA | 25 - 50 µM | [18] |
| Na^+^/ Ca^2+^ exchanger | NCX | NiCl_2_ | NiCl_2_ | 0.5 - 5 mM | [19] |
