# Supplementary figures and images for: NCX1 represents an ionic Na+ sensing mechanism in macrophages
Source: PLoS Biol. 2020 Jun 22;18(6):e3000722. doi: 10.1371/journal.pbio.3000722 (PMC7307728; doi:10.1371/journal.pbio.3000722)

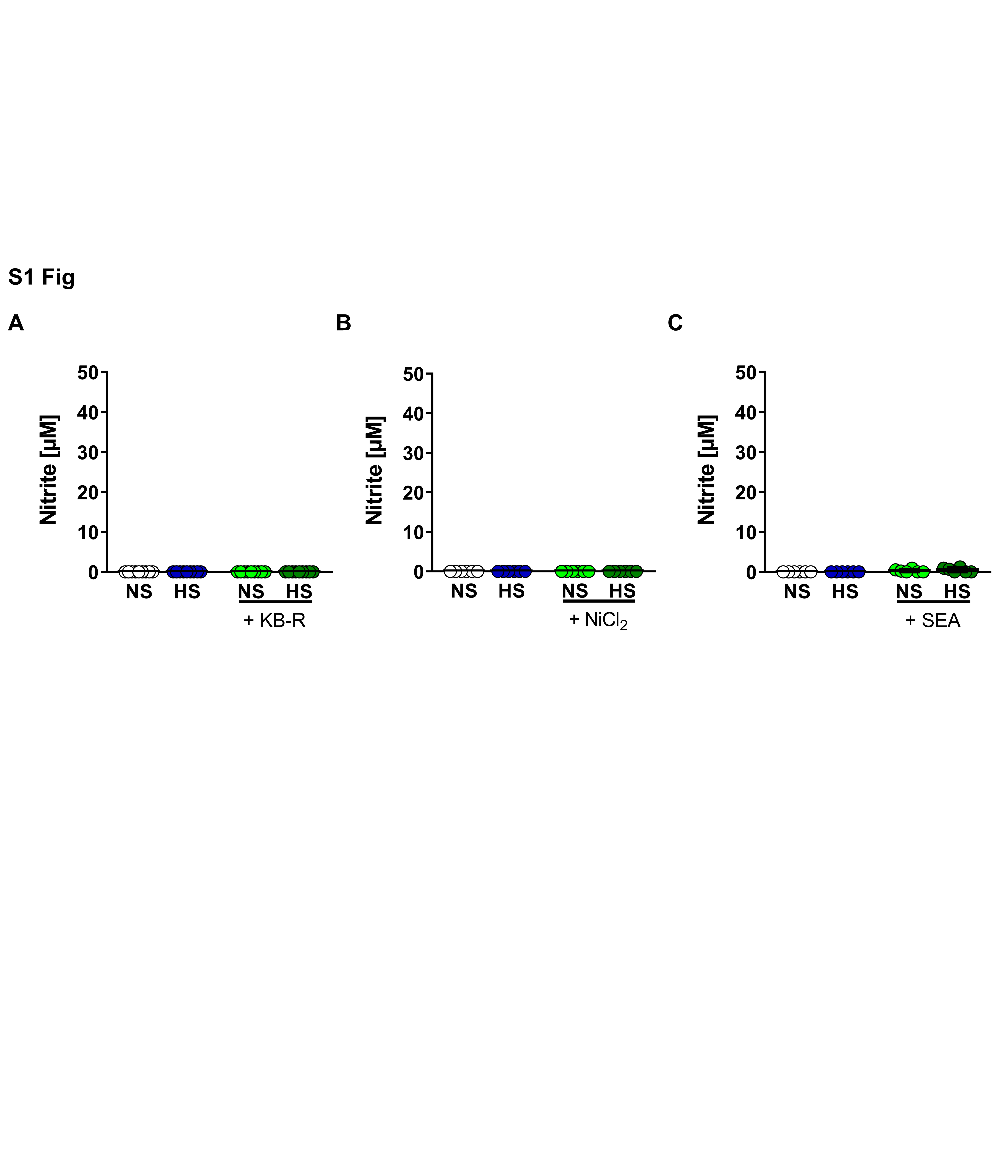

Supplement: S1 Fig — NO production was quantified 24 h after incubation of RAW264.7 MΦs ± HS ± after pretreatment with indicated inhibitor. (A) Inhibitor KB-R (n = 9). (B) Inhibitor NiCl2 (n = 6). (C) Inhibitor SEA (n = 6). For numerical raw data, please see S1 Data. HS, high salt; MΦ, monocyte/macrophage-like cell; KB-R, KB-R7943 mesylate; NCX, Na+/Ca2+ exchanger; NO, nitric oxide; SEA, SEA 0400. (TIF) [file pbio.3000722.s002.tif]

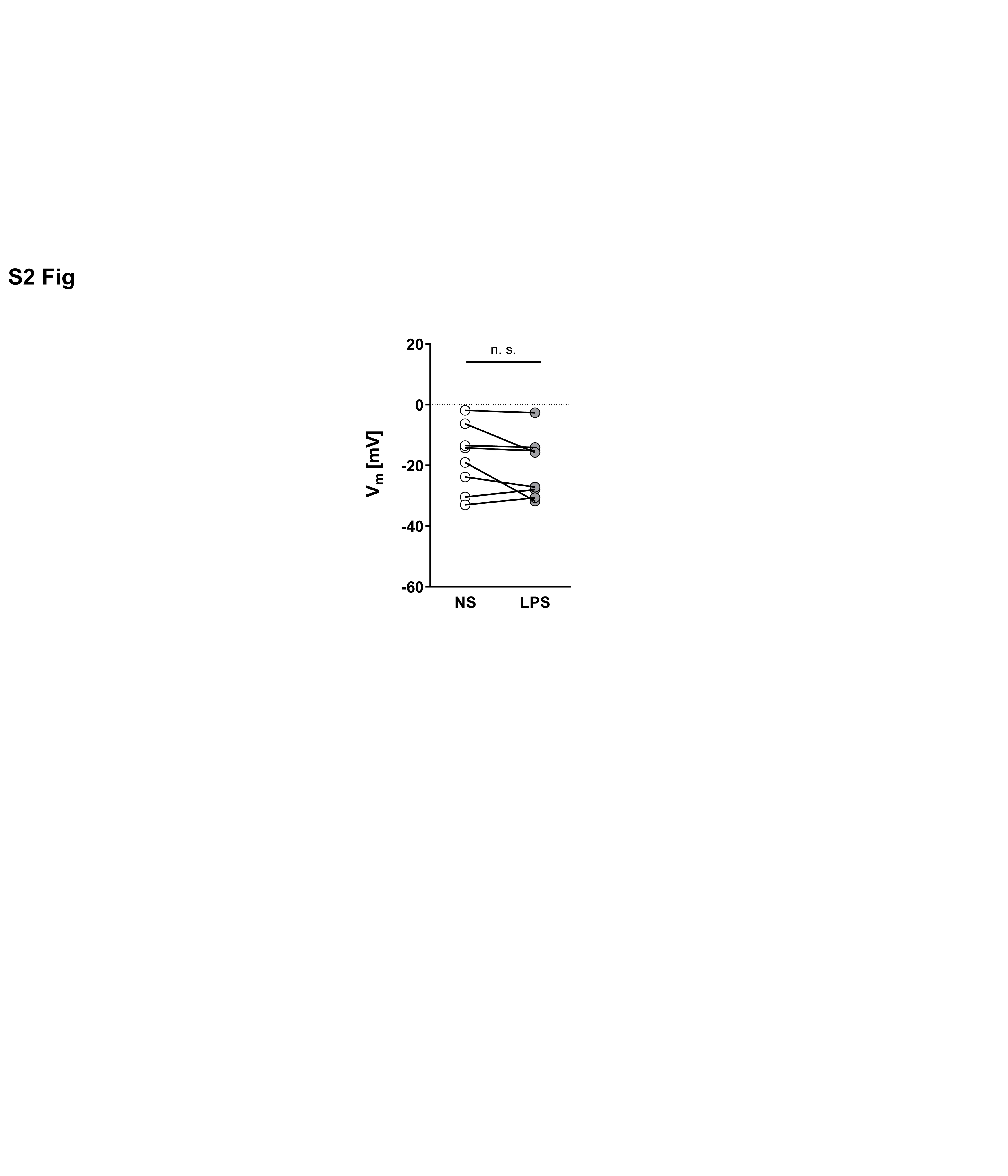

Supplement: S2 Fig — Vm of BMDMs before and after addition of 10 ng/ml LPS (n = 8; paired t test; *p < 0.05). For numerical raw data, please see S1 Data. BMDM, bone marrow–derived MΦ; LPS, lipopolysaccharide; MΦ, monocyte/macrophage-like cell; Vm, membrane potential. (TIF) [file pbio.3000722.s003.tif]

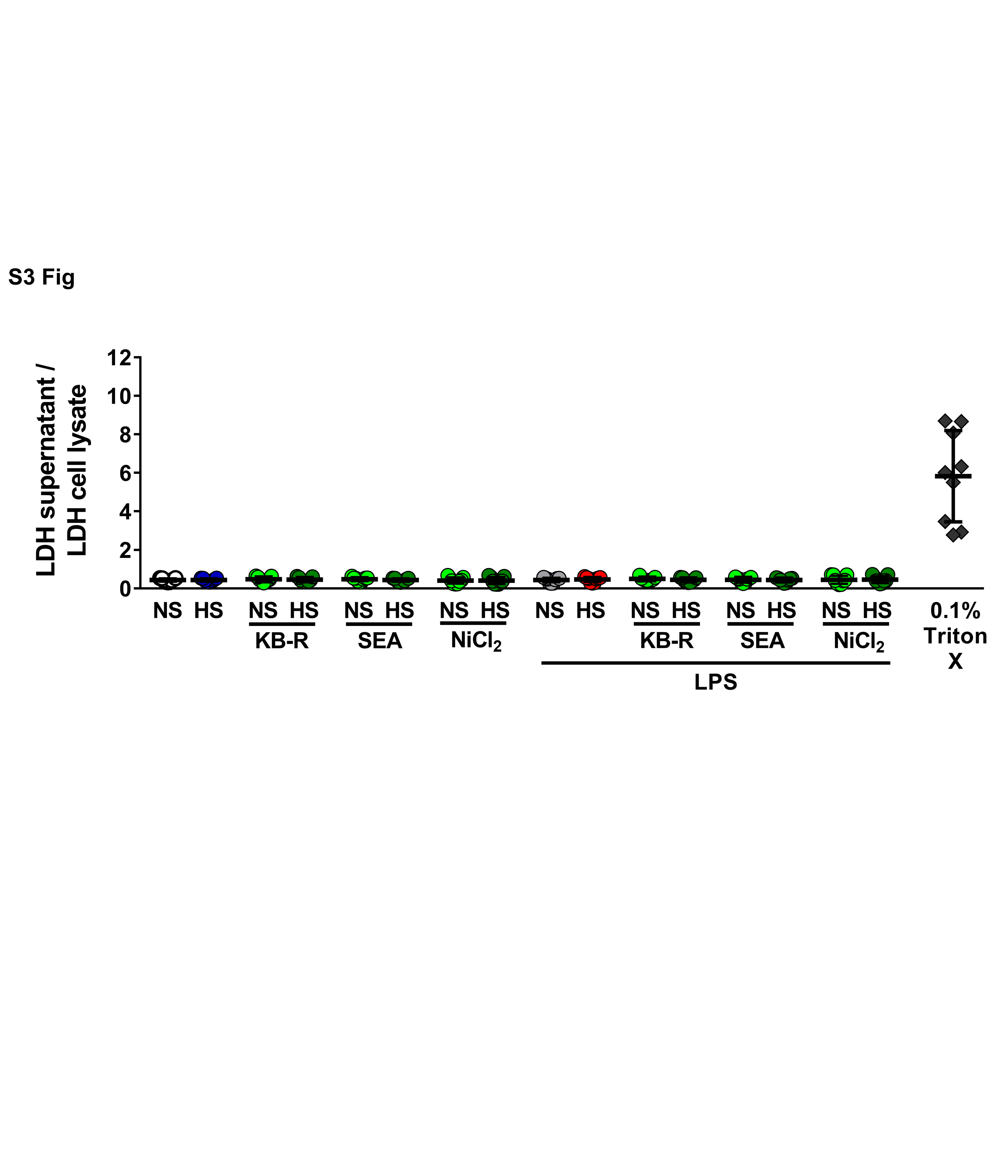

Supplement: S3 Fig — RAW264.7 MΦs were pretreated with indicated NCX inhibitors. At 4 h after stimulation ± LPS ± HS relative LDH release normalized to total cellular LDH content was assessed. Exposure of cells to 0.1% Triton X was used as positive control for cytotoxicity (means ± SD; n = 9). For numerical raw data, please see S1 Data. HS, high salt; LPS, lipopolysaccharide; MΦ, monocyte/macrophage-like cell; NCX, Na+/Ca2+ exchanger. (TIF) [file pbio.3000722.s004.tif]

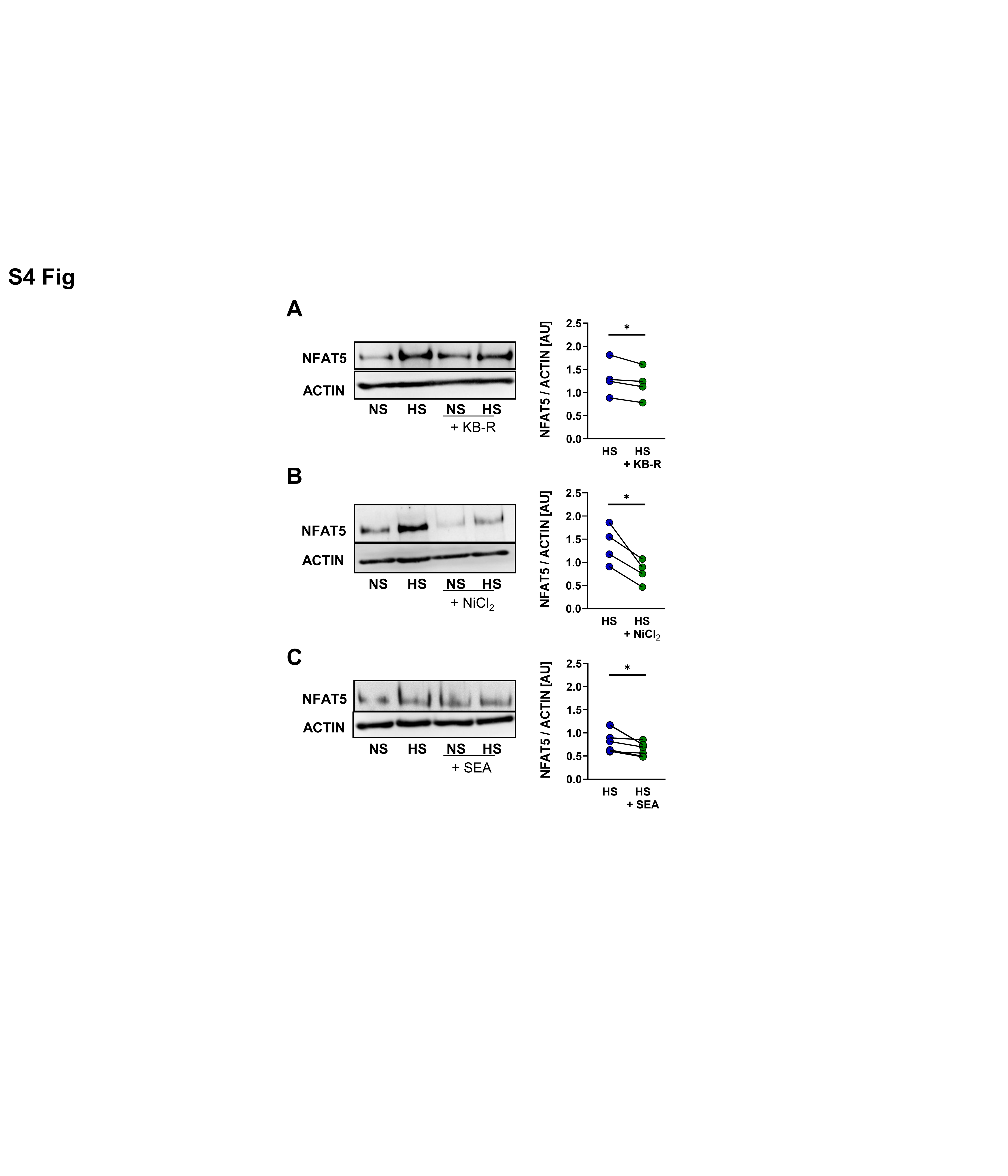

Supplement: S4 Fig — (A) NFAT5 levels 6 h after incubation of RAW264.7 MΦs ± HS ± KB-R pretreatment. Immunoblotting and densitometry (n = 4; paired t tests; *p < 0.05). (B) NFAT5 levels 4 h after incubation of RAW264.7 MΦs ± HS ± NiCl2 pretreatment. Immunoblotting and densitometry (n = 4; paired t tests; *p < 0.05). (C) As in (B), but SEA was used to inhibit NCX (n = 7; paired t tests; *p < 0.05). For numerical raw data, please see S1 Data. For raw immunoblots, please see S1 Blots. HS, high salt; KB-R, KB-R7943 mesylate; MΦ, monocyte/macrophage-like cell; NCX, Na+/Ca2+ exchanger; NFAT5, nuclear factor of activated T cells 5; SEA, SEA 0400. (TIF) [file pbio.3000722.s005.tif]

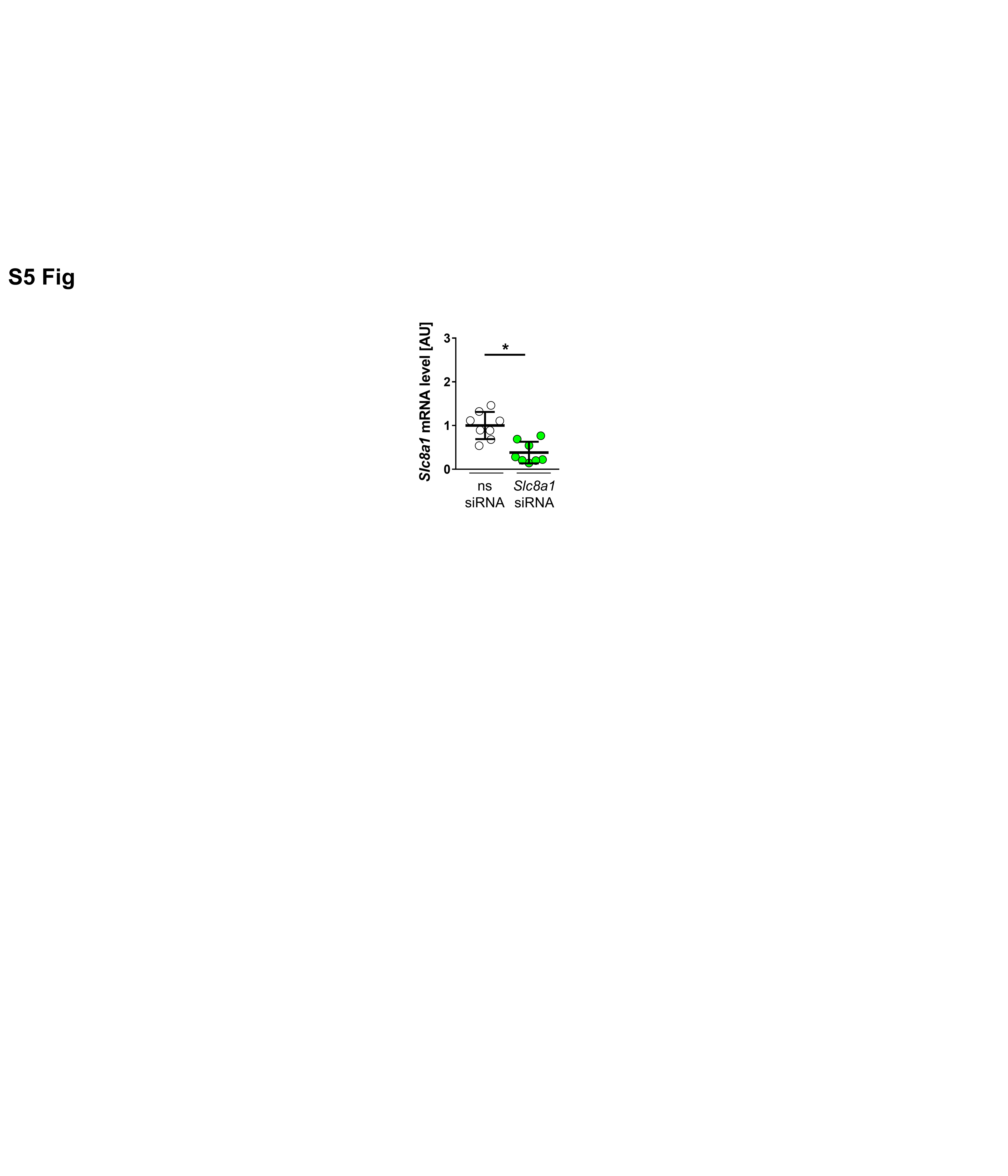

Supplement: S5 Fig — Slc8a1 expression in ns or Slc8a1-specific siRNA–treated BMDMs (means ± SD; n = 8; Student t test; *p < 0.05). For numerical raw data, please see S1 Data. BMDM, bone marrow–derived MΦ; ns, nonsilencing; siRNA, small interfering RNA; Slc8, solute carrier family 8. (TIF) [file pbio.3000722.s006.tif]

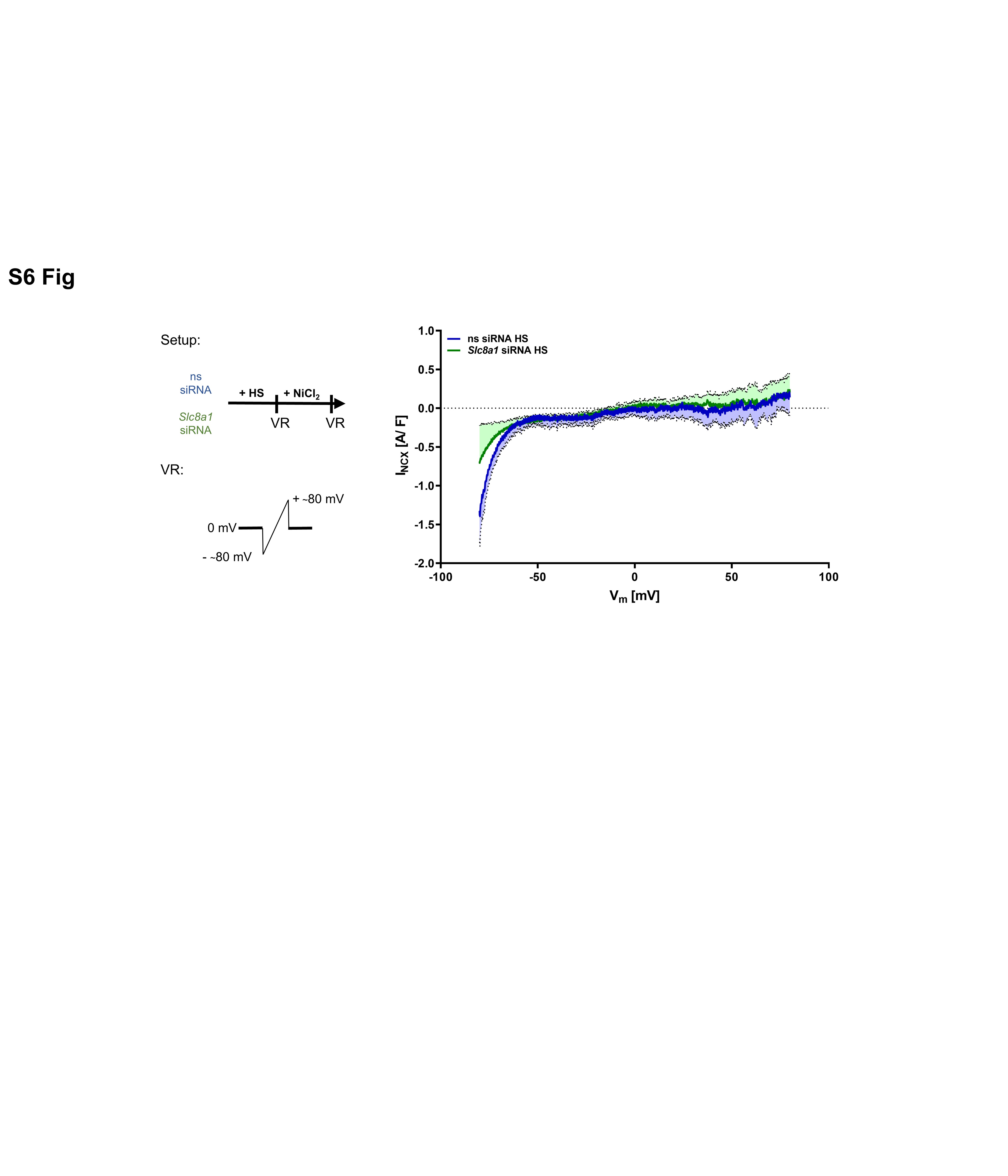

Supplement: S6 Fig — Current/voltage relationships of BMDMs treated with ns siRNA and Slc8a1 siRNA. These MΦ were stimulated with HS followed by NiCl2 treatment. Whole-cell VR experiments were performed, and Ni-sensitive (i.e., NCX-sensitive) currents were determined (means ± 95% CI; n = 9–10). For numerical raw data, please see S1 Data. BMDM, bone marrow–derived MΦ; HS, high salt; MΦ, monocyte/macrophage-like cell; NCX, Na+/Ca2+ exchanger; ns, nonsilencing; siRNA, small interfering RNA; Slc8, solute carrier family 8; VR, voltage ramp. (TIF) [file pbio.3000722.s007.tif]

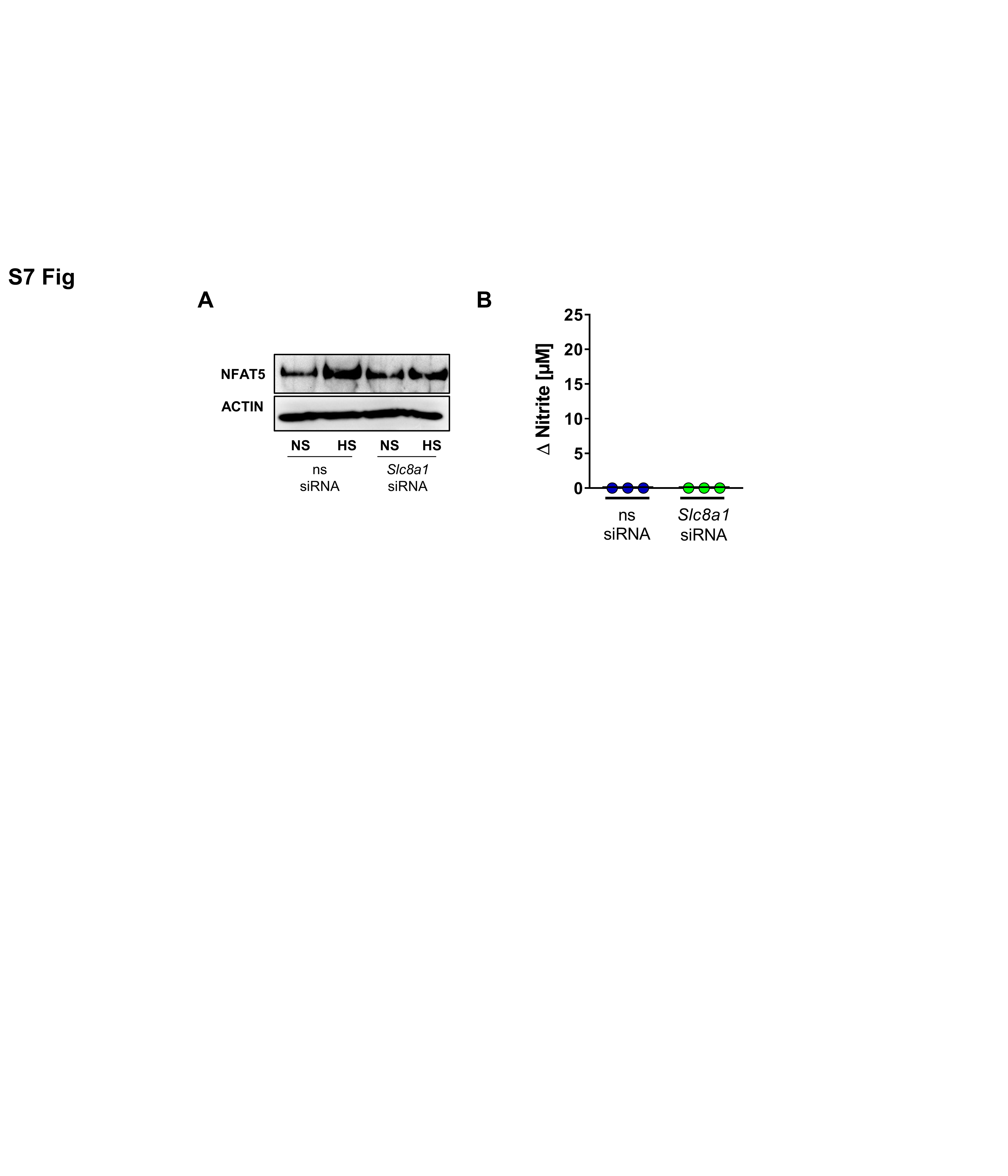

Supplement: S7 Fig — (A) NFAT5 expression 4 h after LPS ± HS in ns siRNA and Slc8a1 siRNA–treated RAW264.7 MΦs. Representative NFAT5 and ACTIN immunoblot out of two similar experiments. (B) As in (A), but HS-triggered Δ nitrite after 24 h (n = 3). For numerical raw data, please see S1 Data. For raw immunoblots, please see S1 Blots. HS, high salt; LPS, lipopolysaccharide; MΦ, monocyte/macrophage-like cell; NFAT5, nuclear factor of activated T cells 5; ns, nonsilencing; siRNA, small interfering RNA; Slc8, solute carrier family 8. (TIF) [file pbio.3000722.s008.tif]
