# Supplementary material for: NCX1 represents an ionic Na+ sensing mechanism in macrophages
Source: PLoS Biol. 2020 Jun 22;18(6):e3000722. doi: 10.1371/journal.pbio.3000722 (PMC7307728; doi:10.1371/journal.pbio.3000722)
Supplement: S1 Blots — The file “S1_Blots.pdf” covers all uncropped western blot images, including size standards and descriptions. (PDF) [file pbio.3000722.s010.pdf]

Fig 7A

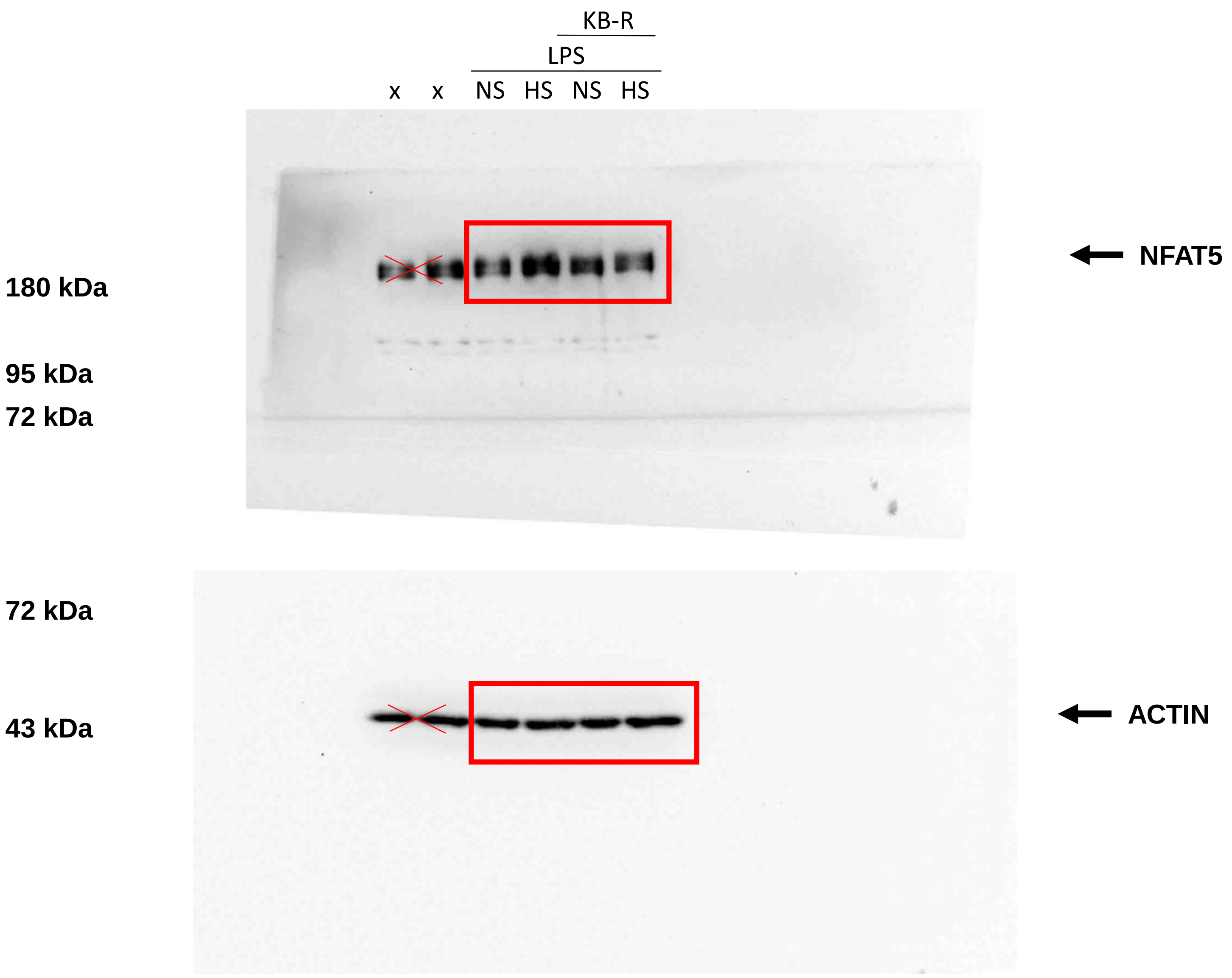
KB-R
LPS
x
x
NS
HS
NS
HS
180 kDa
95 kDa
72 kDa
NFAT5
72 kDa
43 kDa
ACTIN

Fig 7C

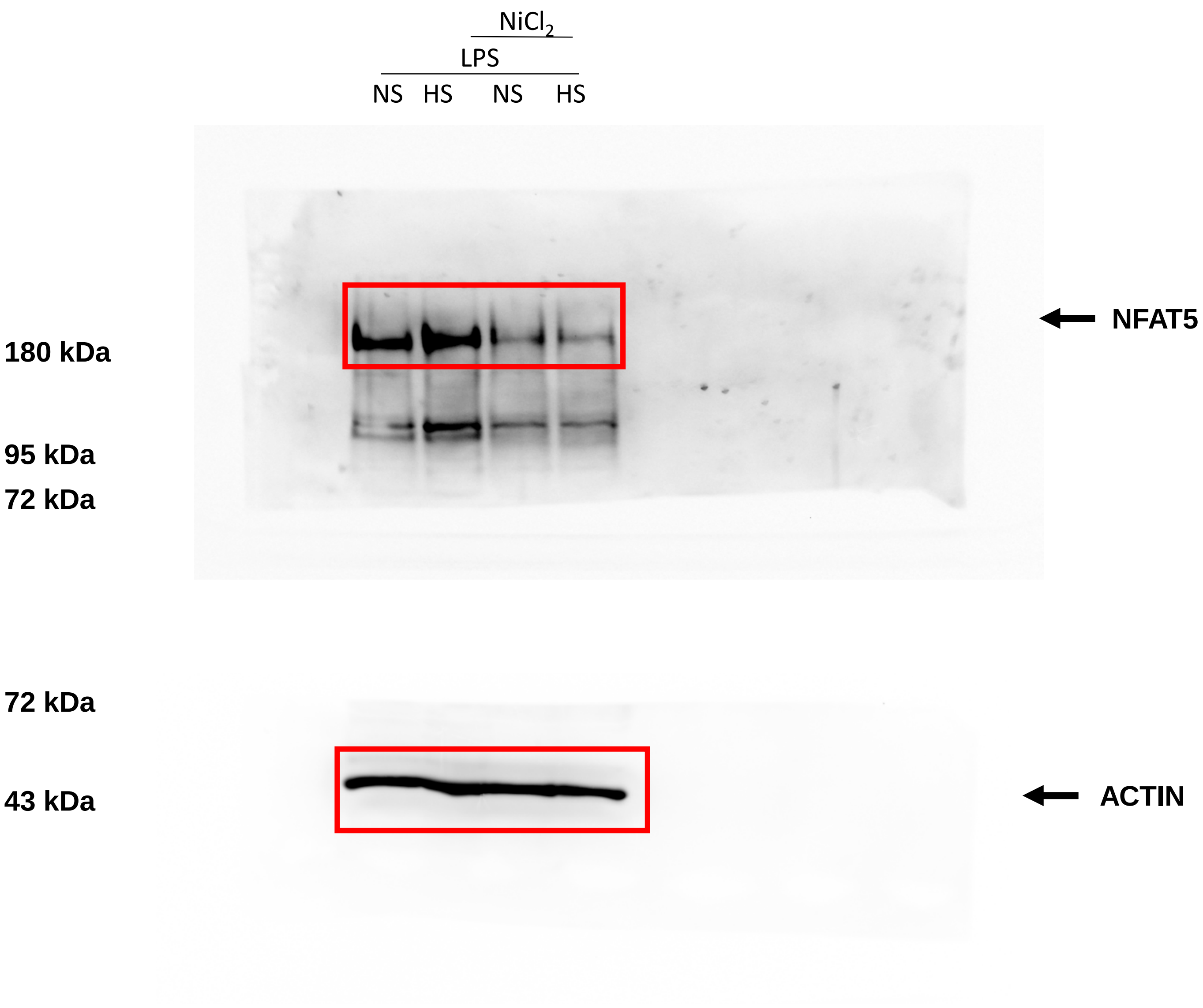
NiCl$_2$
LPS
NS
HS
NS
HS
180 kDa
95 kDa
72 kDa
NFAT5
72 kDa
43 kDa
ACTIN

Fig 7E

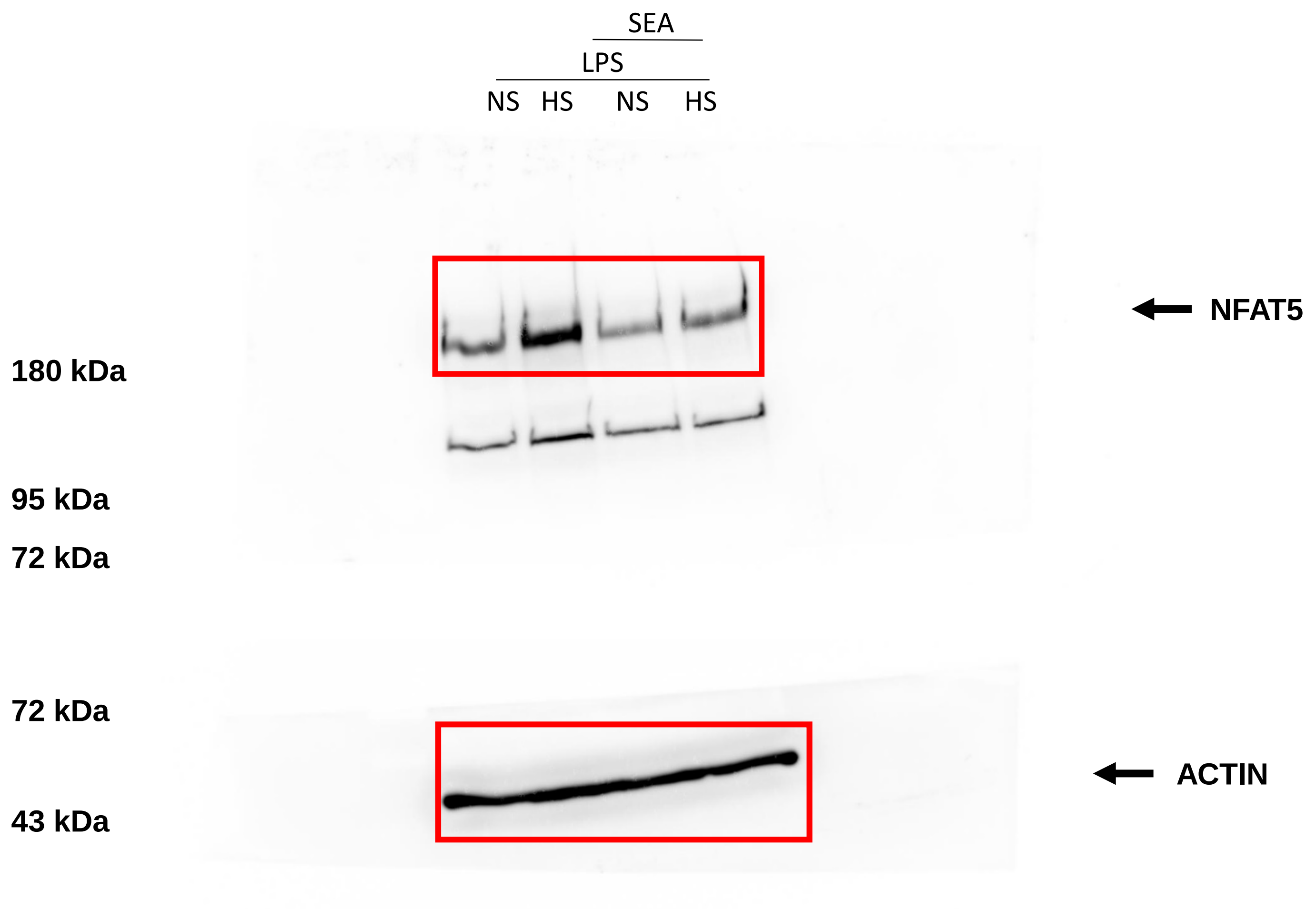
SEA
LPS
NS
HS
NS
HS
180 kDa
95 kDa
72 kDa
NFAT5
72 kDa
43 kDa
ACTIN

Fig 8B

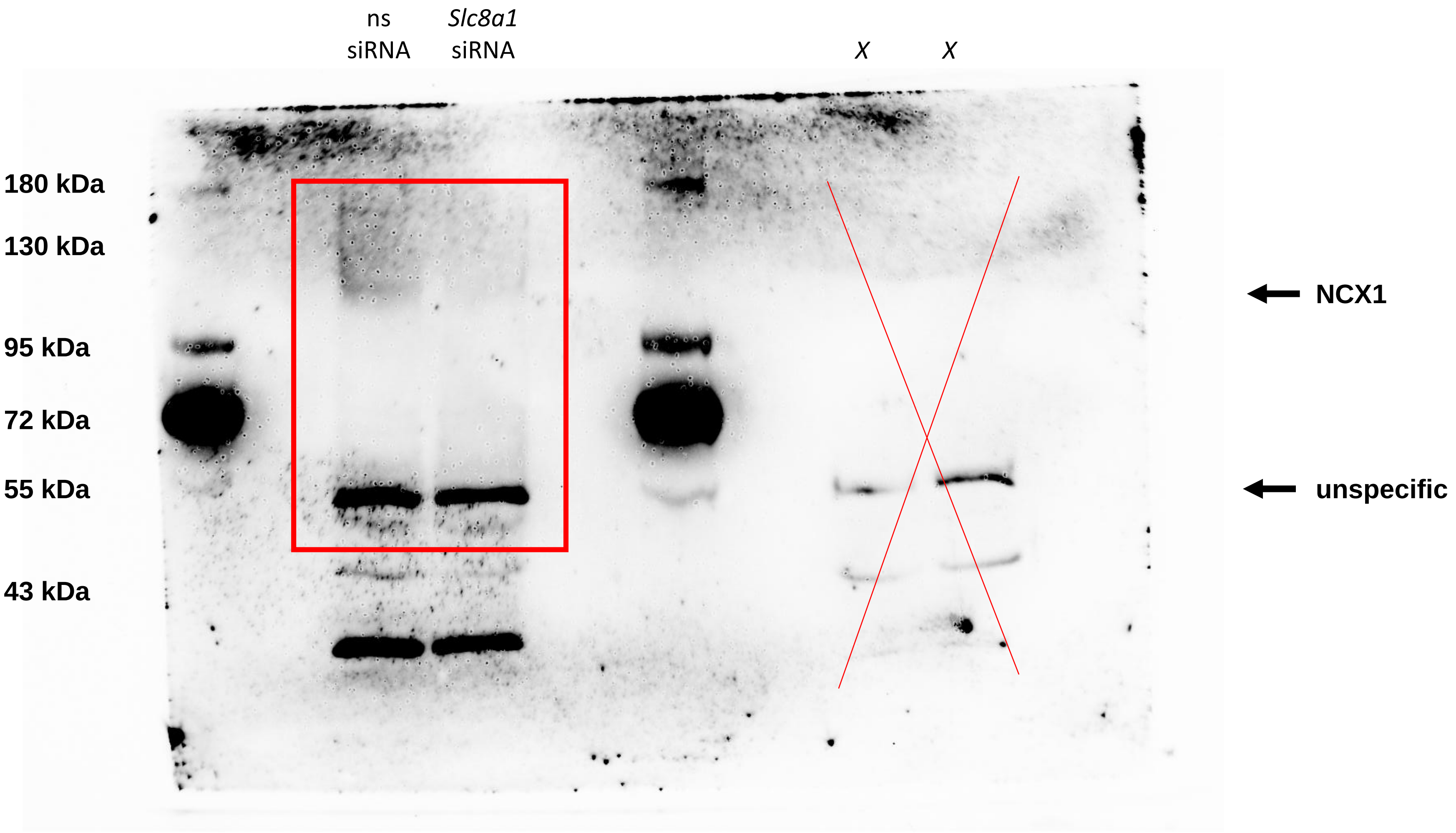

ns siRNA
Slc8a1 siRNA
X
X
180 kDa
130 kDa
95 kDa
72 kDa
55 kDa
43 kDa
NCX1
unspecific

Fig 9A

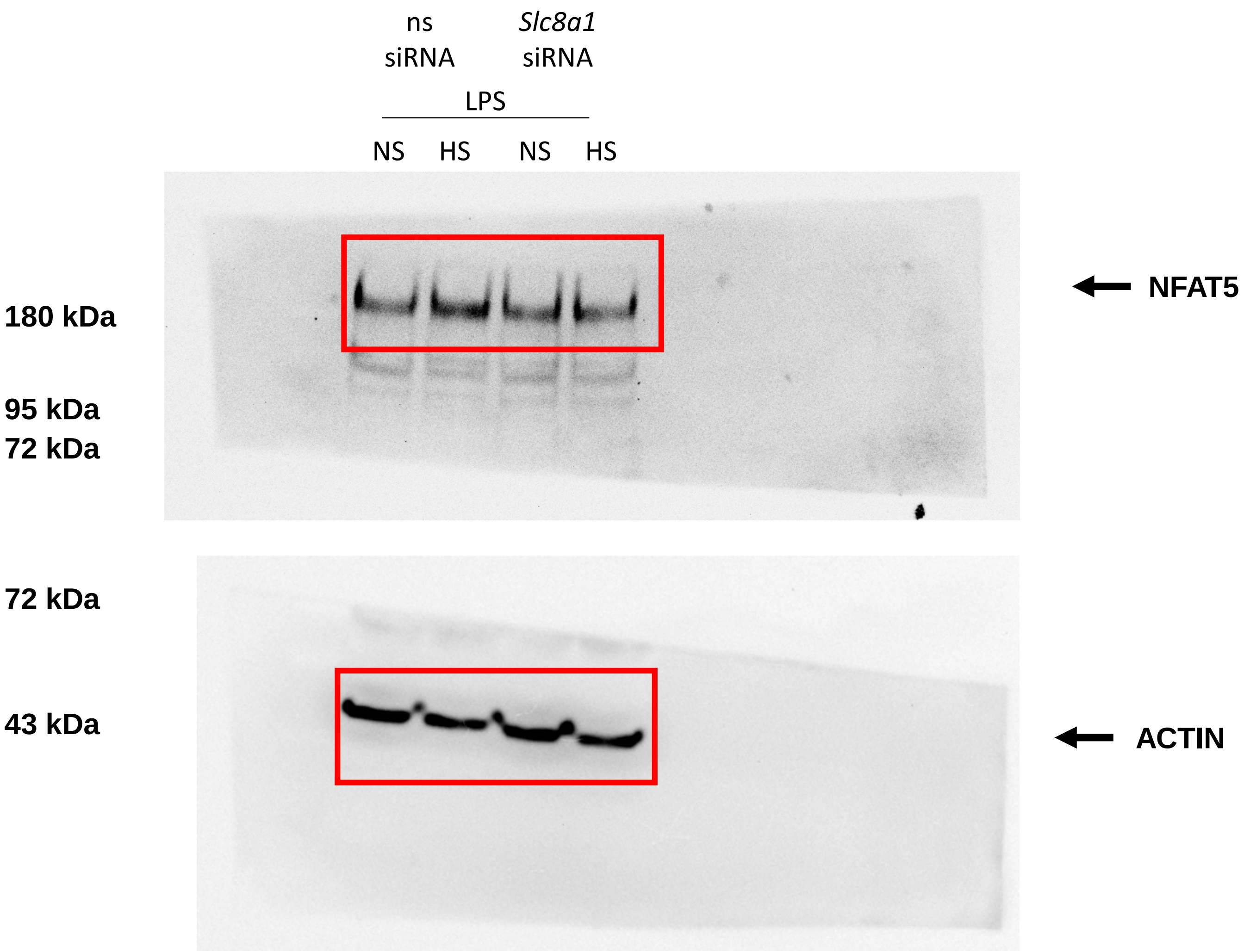
ns
siRNA
Slc8a1
siRNA
LPS
NS
HS
NS
HS
180 kDa
95 kDa
72 kDa
NFAT5
72 kDa
43 kDa
ACTIN

S4A Fig

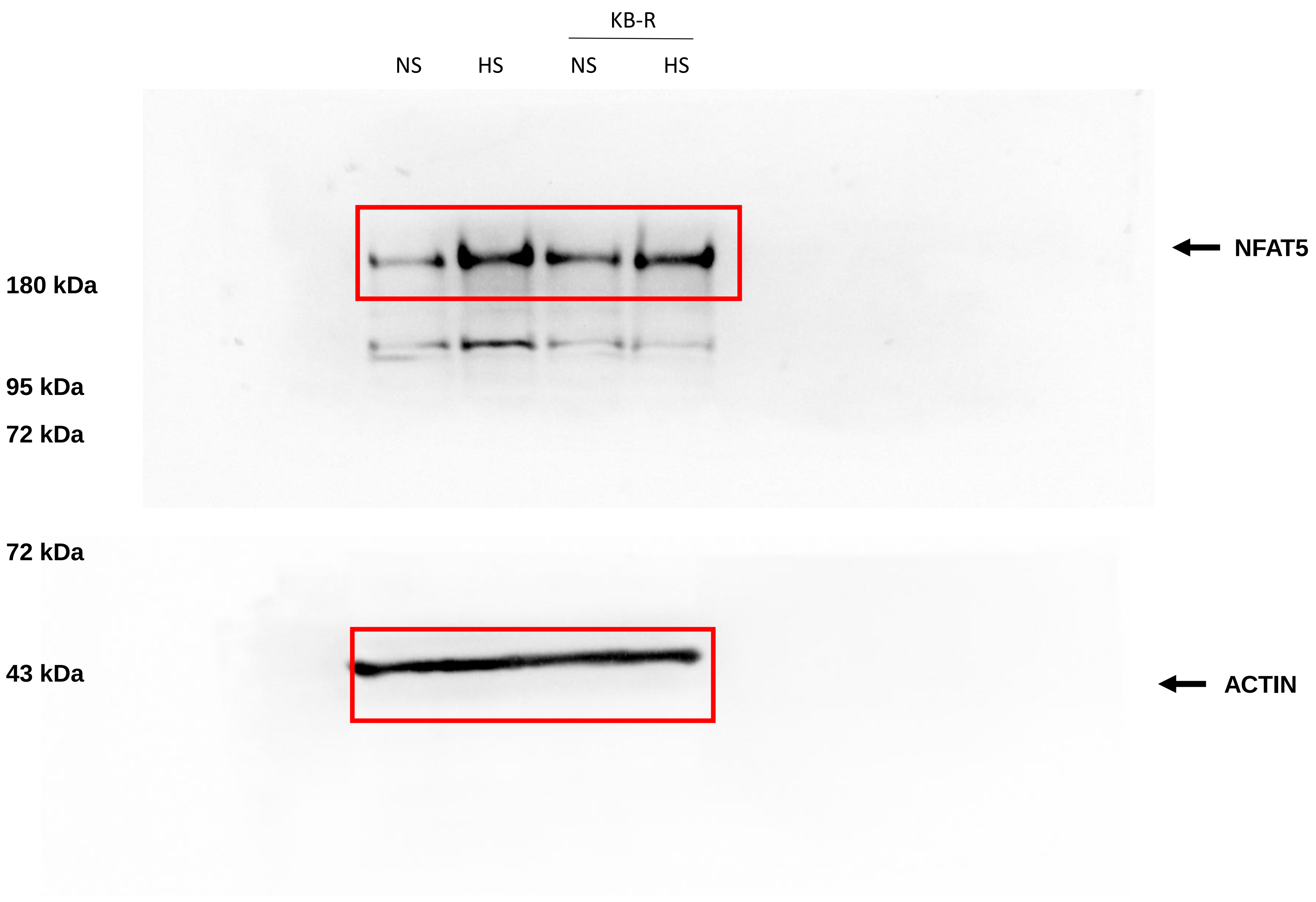

S4B Fig

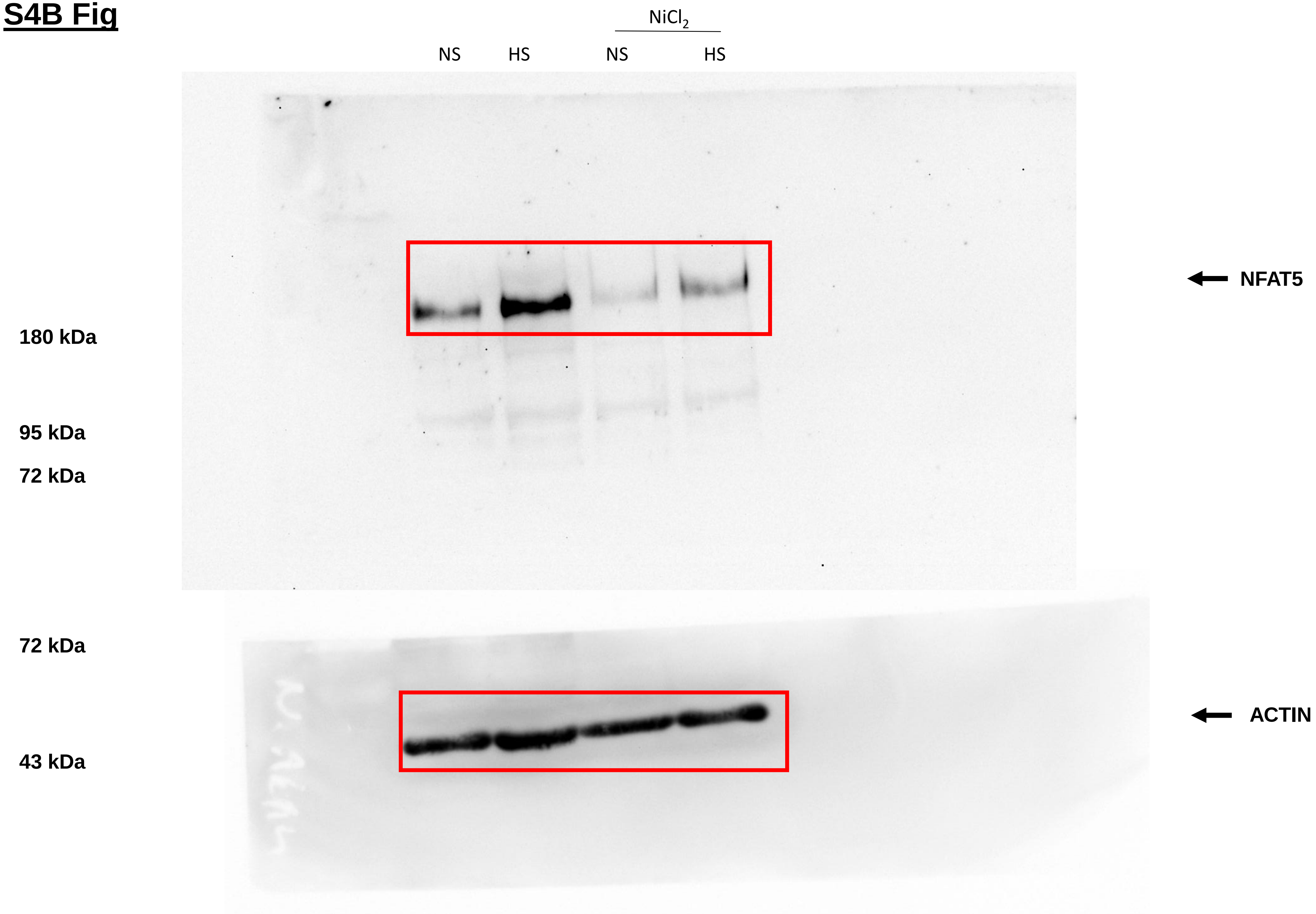

S4C Fig

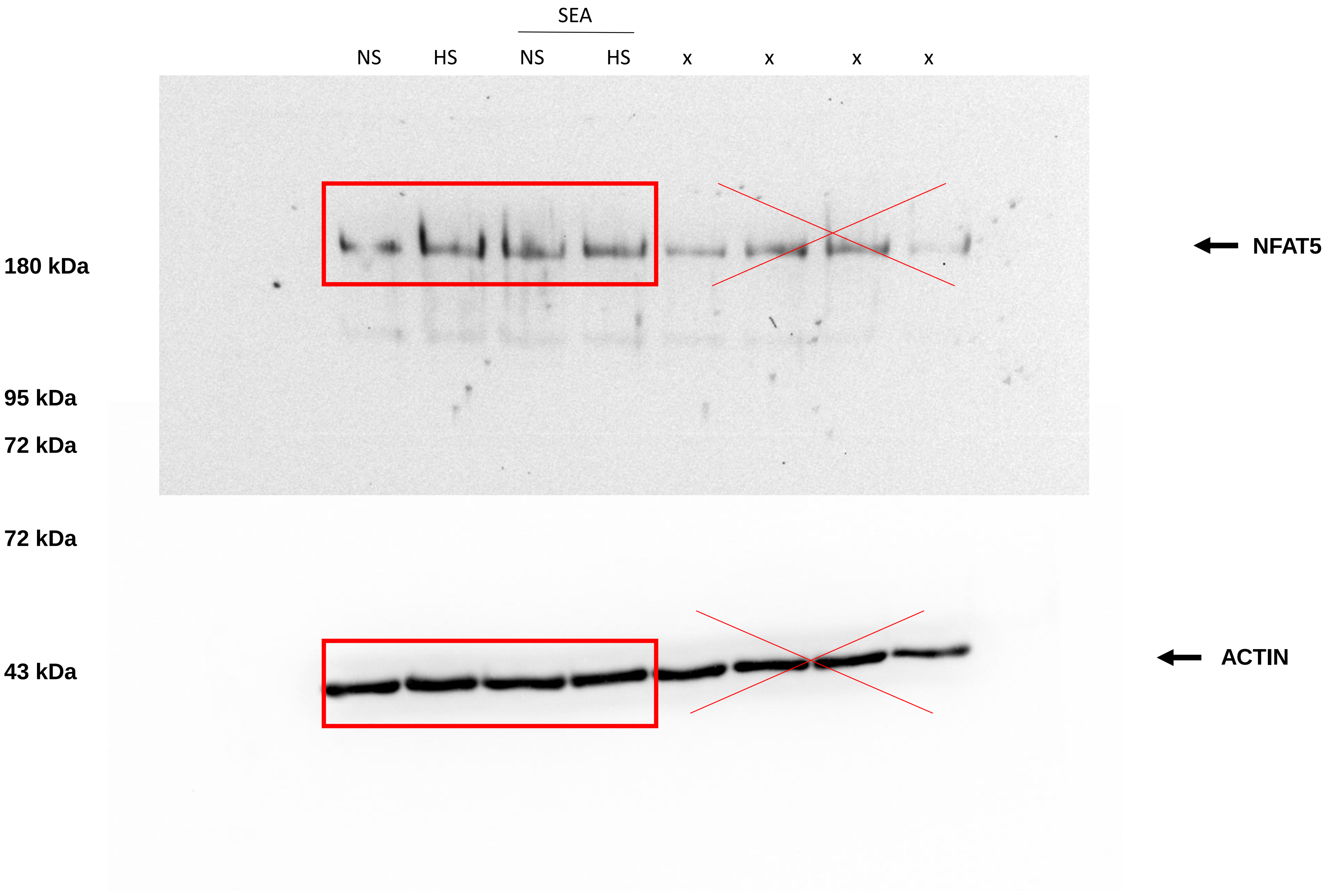

SEA
NS
HS
NS
HS
x
x
x
x
180 kDa
95 kDa
72 kDa
72 kDa
43 kDa
NFAT5
ACTIN

S7A Fig

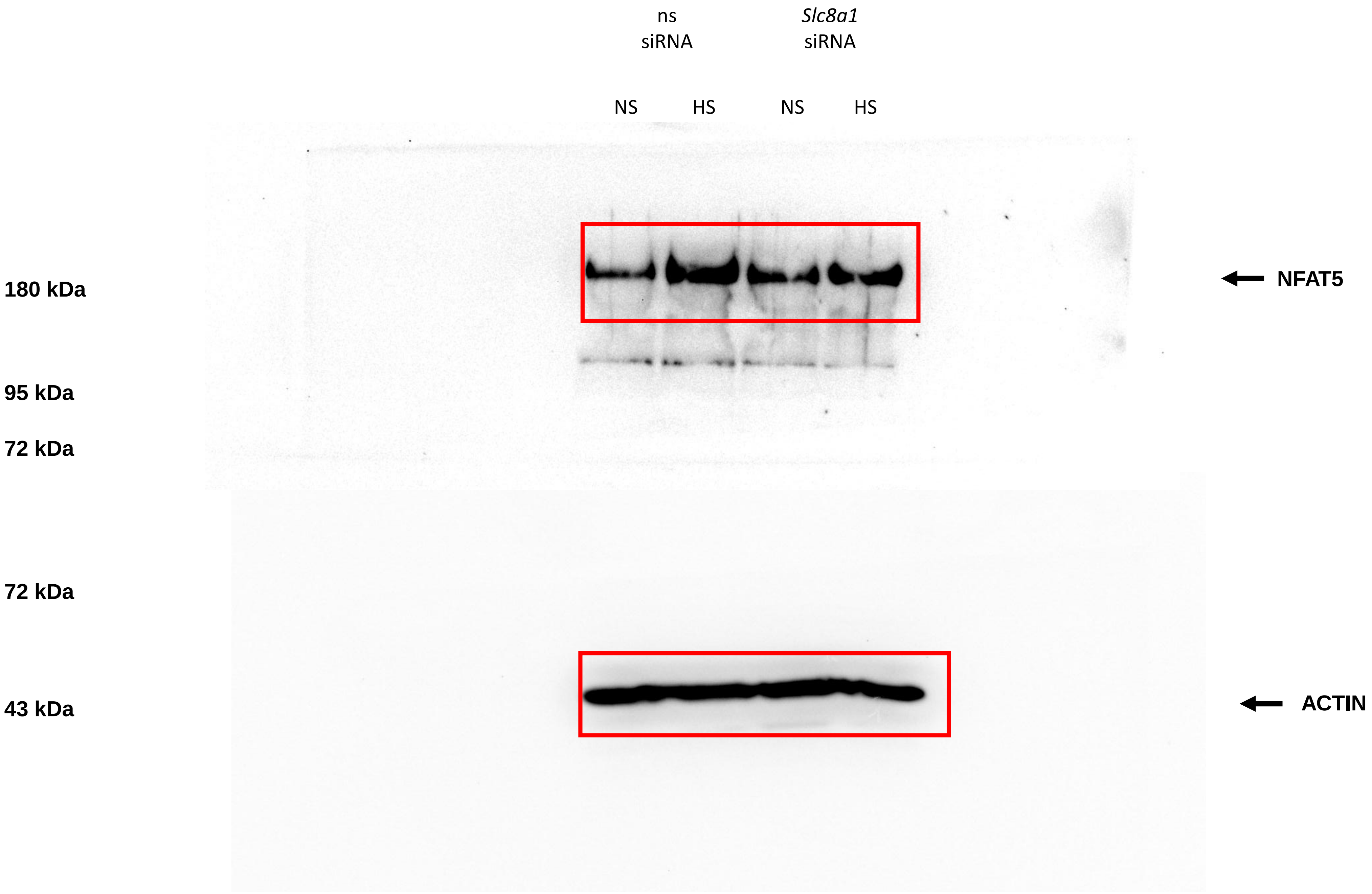

ns
siRNA
Slc8a1
siRNA
NS
HS
NS
HS
180 kDa
95 kDa
72 kDa
72 kDa
43 kDa
NFAT5
ACTIN
